# Pigeons, portals, and Pacman: Insightful problem solving and navigation using a touchscreen video game

**DOI:** 10.1002/jeab.70083

**Published:** 2026-02-01

**Authors:** Rafael S. Rodrigues, Cyrus Kirkman, Miriam Garcia‐Mijares, Aaron P. Blaisdell

**Affiliations:** ^1^ Department of Experimental Psychology, Institute of Psychology University of São Paulo São Paulo Brazil; ^2^ Department of Psychology University of California, Los Angeles Los Angeles CA USA

**Keywords:** insight, pigeons, video game

## Abstract

Video games have been used in several studies to investigate problem solving. We present empirical findings from a redesigned touchscreen navigation “grid‐world” procedure resembling the classic video game PacMan (hereafter, “pacman”) played by pigeons. Our objective was to develop a procedure to study insight, similar to that of Epstein et al. (1984). During Training Phase 1, pigeons learned to guide a virtual pacman across a touchscreen arena to a banana goal (triggering an “eating” animation and food delivery) and to navigate around barriers. In Training Phase 2, they learned to traverse portals that transported the pacman across the arena. Each tool was trained in a distinct context: barrier navigation with banana targets and portal use with a green dot target. Finally, we tested for functional generalization of the two learned tools in a series of novel configurations of puzzle tasks (insight tests). Results showed that some subjects were able to learn how to navigate the pacman with a high degree of control and showed evidence for functional generalization and insight.

A snake spots a lizard and attempts to catch and eat it. During the snake's pursuit, the lizard climbs a nearby tree. The snake changes its approach. Instead of directly pursuing the lizard, the snake looks around and spots a nearby tree with overhanging branches, which it climbs. While the lizard faces downward toward the ground—looking where its pursuer had been—the snake climbs this other tree to a higher elevation than the lizard. The snake proceeds to extend itself along a branch and toward the lizard's tree. With a sudden strike, the snake knocks the lizard to the ground. The snake quickly drops after its prey and easily catches the stunned lizard. This anecdote—reported by Charles Henry Turner in 1909—is perhaps the first scientific report of problem‐solving behavior by a wild animal that seemed directed and sudden (Turner, 1909). Turner recognized that the snake's behavior likely did not involve a “mindless” process such as instincts or habits or trial‐and‐error learning during the attempt to catch the lizard. Rather, he thought that the behavior was specific to this situation and emerged in full form without deliberation or prior unsuccessful attempts. Nevertheless, without knowing the history of the snake, one cannot rule out prior hunting experiences where the snake may have learned through trial‐and‐error processes involving reinforcement and nonreinforcement to engage in this behavior. Turner himself acknowledged the limitations of this anecdote from the wild and yet also recognized the many interesting questions that this anecdote raises.

Just over a decade later, Wolfgang Köhler (1925/1959) published his extensive demonstrations of problem solving in chimpanzees. Köhler presented chimpanzees with novel problems, such as suspending a banana out of reach in their outdoor enclosure. Although most of the chimps persisted in their unsuccessful attempts to grasp the banana by reaching up toward it, one chimp named Sultan gave up those attempts and instead dragged shipping crates over to where the banana was, stacked the crates on top of each other, and then climbed the crates to retrieve the banana. Köhler interpreted this as Sultan having insight into the solution to the problem. He contrasted such insight with the trial‐and‐error learning Thorndike's (1898) cats showed in learning to solve a problem, such as escaping from a puzzle box, which we discuss next.

In his classic experiment, Thorndike (1898) placed a cat in a wooden cage, the door of which could be released by a tug on a latchstring. Prior to this, the cat had never seen the latchstring. It initially tried to squeeze through the bars and claw and bite at the bars. As these behaviors were unsuccessful in releasing the cat from the cage, they were extinguished during the trial. Eventually, the cat happened to claw at the loop on the latchstring and pull it, thereby successfully opening the door. The cat received repeated trials of this sort, and the prepotent responses of biting, scratching, and pushing at the bars declined while the behavior of pulling the latchstring became more prominent, resulting in progressively shorter escape times. Based on this and related experiments, Thorndike (1898) proposed that the acquisition of new behaviors, especially those that solved a problem (e.g., release from captivity) could be explained in terms of trial‐and‐error learning resulting in stimulus–response associations.

According to Köhler (1925/1959), however, Thorndike's animals only exhibited this gradual acquisition of a new response because they did not easily perceive the functional relations involved between the action and outcome. For example, in a puzzle box in which pulling a latchstring leads to escaping from the cage, there is no readily apparent functional relation between the string and the cage (Birch, 1945; Shettleworth, 2012). Thus, by constructing problem situations where these functional relations were more evident, Köhler argued that another type of solution could appear: an “insightful” solution. In the example of the banana‐and‐box problem described above, Köhler advocated for a distinction between solving a problem gradually through successive approximations (present in Thorndike's cats) versus through insight—that is, suddenly and continuously (as in the performances shown by Sultan). He claimed that insight is due to the animal suddenly realizing the solution to the problem (Shettleworth, 2012).

Since these early demonstrations, insight has continued to receive empirical attention, largely in the fields of human cognition and neuroscience. In a classic human study of insight, human participants were provided with a candle, matches, and a box of thumbtacks and asked to fix a lit candle to the wall (Duncker & Lees, 1945). An effective solution to this puzzle is to attach the box to the wall with a few tacks, using the box as a shelf or ledge on which the candle can be placed. To find this solution, it is necessary to perceive the box as serving a function other than simply holding tacks, overcoming what Duncker and Lees called “functional fixation,” or the tendency to categorize objects as serving specific functions in a way that limits applications to alternative uses. This flexibility of discovering alternative uses of a categorized object is a phenomenon that Gestalt psychologists labeled as “perceptual restructuring” (Shettleworth, 2012), which they deemed a prerequisite for insight to occur. Thus, unlike trial‐and‐error learning, which happens gradually, insight is characterized by the typically rapid, untrained occurrence of a new functional relation that results in a single and ordered behavioral chain (Santana & Garcia‐Mijares, 2022) in which a subject does not gradually arrive at a solution by successive approximations but instead by exhibiting the behavioral chain that solves the problem all at once (Shettleworth, 2012).

Despite its early origins in the study of nonhuman animals, insight has received fairly limited attention in the field of comparative psychology (Osuna‐Mascaró & Auersperg, 2021). Most animal work on insight involves tool‐use tasks (Call, 2013). One example involves an adaptation of Köhler's banana‐and‐box task for use with pigeons. Epstein et al. (1984) reinforced pigeons' responses with food when pecking at a small plastic toy banana. Then, they reinforced climbing on a box and pecking the banana when the box was fixed in place under the banana. Flying behavior was also extinguished. Pigeons also underwent training in which the banana was absent and pigeons' responses were instead reinforced when pushing a movable box toward a green spot located on a wall of the enclosure. The location of the green spot varied across trials such that moving the box was under the control of the green spot stimulus. Finally, pigeons were presented with a novel situation with the suspended banana and a moveable box but no spot on the wall. Initially, pigeons looked back and forth between the banana and the box. Within a couple of minutes, however, they abruptly began to push the box in the direction of the banana, stopped when the box was positioned underneath the banana, and then climbed the box to peck at the banana. According to Epstein et al. (1984) and Epstein (1985a), these pigeons showed insight by generalizing the function of the green spot (i.e., indicating the direction the box should be moved) to the banana, a phenomenon that he called functional generalization. Later, Shettleworth (2012) interpreted those results as mediated generalization, a form of generalization distinguished by the transfer of discriminative function between physically distinct stimuli resulting from their shared relation with other events (Urcuioli, 2006).

If insight reflects mediated generalization, altering the learned relation should affect functional transfer. Accordingly, a replication by Luciano (1991) trained two pigeons to push the box *away* from the green spot. Whereas one pigeon pushed the box away from the banana on the final insight test, the other pigeon did not push the box at all. This suggests that there is indeed functional transfer of the pushing response from the green spot to the banana as the target of pushing, with transfer being stronger when the function was the same across both (e.g., “pushing toward” both banana and spot) than when functional behavior was in conflict (e.g., “pushing toward” banana vs. “pushing away” from spot).

A second notable feature of the Epstein et al. (1984) study is that the order in which the trained behaviors emerged at test depended on the starting conditions at test. Initially on the test trial, the banana was suspended out of reach, which was a condition during training in which flying toward the banana had been extinguished. A moveable box was also present, however, which replicated the stimulus conditions during training to push the box toward the spot. The latter stimulus condition was closest to prior training, and thus box pushing was initially controlled by the banana at test. Once the box was under the banana, this stimulus condition mirrored that during training to stand on the box and peck the banana—which is precisely what the pigeons then did. Epstein (1985b) referred to this orderly emergence of separately trained behaviors as autochaining (short for automatic chaining). Epstein et al. (1984) proposed that insight could be fully explained through the processes of functional generalization and autochaining. Autochaining has since been demonstrated with the interconnection of longer behavioral sequences (Epstein, 1985b, 1987) and in other species such as rats (Neves Filho et al., 2015), capuchin monkeys (Neves Filho et al., 2016), and crows (Neves Filho et al., 2019). We revisit discussion of these processes in the Discussion.

Currently, all procedures used to study insight in animals use real‐world situations involving observations of behavior on critical test trials. This presents several problems that impede progress in understanding and assessing insight in animals. Real‐world settings are often complex and unpredictable and lead to a lack of control over the variables that may be influencing insight. Furthermore, because the data are generally observational in nature and molar behaviors of freely moving organisms are often challenging to quantify, it is difficult to make precise interpretations of the behavior at test aside from latency to solve the problem or types of actions observed under test conditions. For example, many of Epstein et al.'s (1984) findings involve observations that the pigeon “looked back and forth” between the box and the banana. Although descriptive, it is difficult to interpret the behavior aside from what it “appears” to be to the observer, and that is often in reference to similar behaviors that might be observed in people and, thus, can suffer from anthropomorphism. Other challenges include standardization of tasks, controlling confounding variables, and difficulty in replicating the procedures across labs. These shortcomings could be remedied through the development of computer‐based tasks using virtual environments. Indeed, video games have a long history of use in the study of human insight and have also been extensively used to study learning and cognition in animals (Seitz et al., 2021). Video‐game procedures have also been used to study problem‐solving behavior in humans and have shown great utility for cognitive and behavioral researchers in general, as they allow better measurement and quantification of what the subjects are doing (e.g., Rodrigues & Garcia‐Mijares, 2021; Sturz et al., 2009). Development of a video‐game environment for studying insight in animals offers several benefits relative to real‐world environments. The following are some advantages of using a video‐game environment:

1. *Controlled Variables*: In a video game, researchers can manipulate variables more precisely, ensuring consistent conditions across trials. This control can lead to clearer identification of the factors affecting insight.
2. *Isolation of Factors*: Video games enable researchers to isolate specific factors and variables to examine their influence on insight, without the potential confounding factors present in real‐world settings.
3. *Reproducibility*: Video‐game tasks can be replicated easily and consistently, allowing other researchers to replicate the experiments and validate the findings.
4. *Efficiency*: Conducting experiments in a virtual environment is often quicker and more efficient than setting up complex real‐world scenarios. This efficiency allows for larger sample sizes and more comprehensive analyses.
5. *Standardization*: Virtual tasks provide a standardized experience for all participants, reducing individual differences that can influence results.
6. *Flexibility*: Researchers can design and modify virtual tasks more easily than real‐world setups, allowing for rapid exploration of various experimental conditions.

If a video‐game task were to be developed to study insight in animals, the benefits would extend further. For example, researchers could design tasks that require different types of behaviors (e.g., escaping a maze, moving a distant object, or solving a jigsaw puzzle), allowing for a deeper understanding of the behavioral processes around insight. Also, video‐game environments can gradually increase task complexity, enabling researchers to systematically investigate how animals handle varying degrees of difficulty. Furthermore, virtual tasks can be combined with neuroimaging techniques to directly observe brain activity associated with insight, shedding light on neural mechanisms. Using similar virtual tasks across multiple species enables direct comparisons, potentially revealing commonalities and differences in insight abilities. Adapting tasks for more direct comparisons between human and nonhuman animals would be especially advantageous (e.g., Racey et al., 2011).

Our goal in the current study was to develop a video‐game‐style approach to studying insight in the pigeon. We specifically attempted a conceptual replication of the banana‐and‐box task used by Epstein et al. (1984), which was itself an adaptation of one of the insight tasks Köhler (1925/1959) gave to his chimpanzees. This approach can enable progress in understanding behavioral processes that contribute to the emergence of insight when pigeons are used as subjects. The quantitative data produced through this procedure will allow for more precise assessments of the different behavioral processes shown by organisms when solving a problem, ranging from Thorndike's trial‐and‐error process to Köhler's sudden insight.

In the current study, we adapted a virtual‐navigation procedure originally developed by Miyata et al. (2006) and Miyata & Fujita (2012) in which pigeons learned to navigate a target across the two‐dimensional (2D) surface of a touchscreen display to reach a visual goal. In Miyata et al. (2006), pecking at a red square displayed on a touchscreen (the target) was first reinforced with food. Once this basic pecking response was established, pigeons were then trained to peck one of four directional guides that appeared around the target (north, east, south, and west). Pecking at one of these guides moved the target in the corresponding direction (e.g., pecking at the west guide moved the target a fixed distance to the west). Then, pigeons were trained to move the target toward a blue square (the goal) by pecking on the correct guides. After this training, pigeons were exposed to a variety of detour tasks in which visual lines drawn on the screen served as barriers to target movement. Pigeons learned these behaviors and solved detour problems. Furthermore, pigeons increasingly chose efficient routes as the barriers gradually became more complex.

Our objective was to use a virtual‐navigation task similar to that of Miyata and Fujita (2012) but with the specific goal of developing and testing a procedure to study insight, following the Epstein et al. (1984) approach. First, we trained the pigeons to guide a virtual pacman (i.e. digital pacman icon) across a virtual arena on a touchscreen display (Experiment 1). When the pacman reached the goal—a visual icon of a banana—the pacman “ate” it and the pigeon's behavior was reinforced through food delivery. Next, we trained pigeons to use and discriminate the utility of two different virtual tools: barriers that had to be circumnavigated to reach the goal (Training Phase 1) and portals that transported the pacman from one side of the arena to another (Training Phase 2). The functions of the tools were trained in different contexts, using either a banana or a green dot icon as the navigational target, respectively. Finally, we tested for functional generalization of the two learned tools in a series of novel configurations of puzzle tasks (insight tests). Figure 1 illustrates how these experiments progressed. Results showed that some pigeons were able to learn how to navigate the pacman with a high degree of control and showed evidence for functional generalization and insight. We discuss how this virtual procedure can be applied to study other problem‐solving tasks and argue for the applicability of this procedure for other animal species, thus allowing for unique and highly rigorous comparative study of problem‐solving phenomena.

**FIGURE 1:**
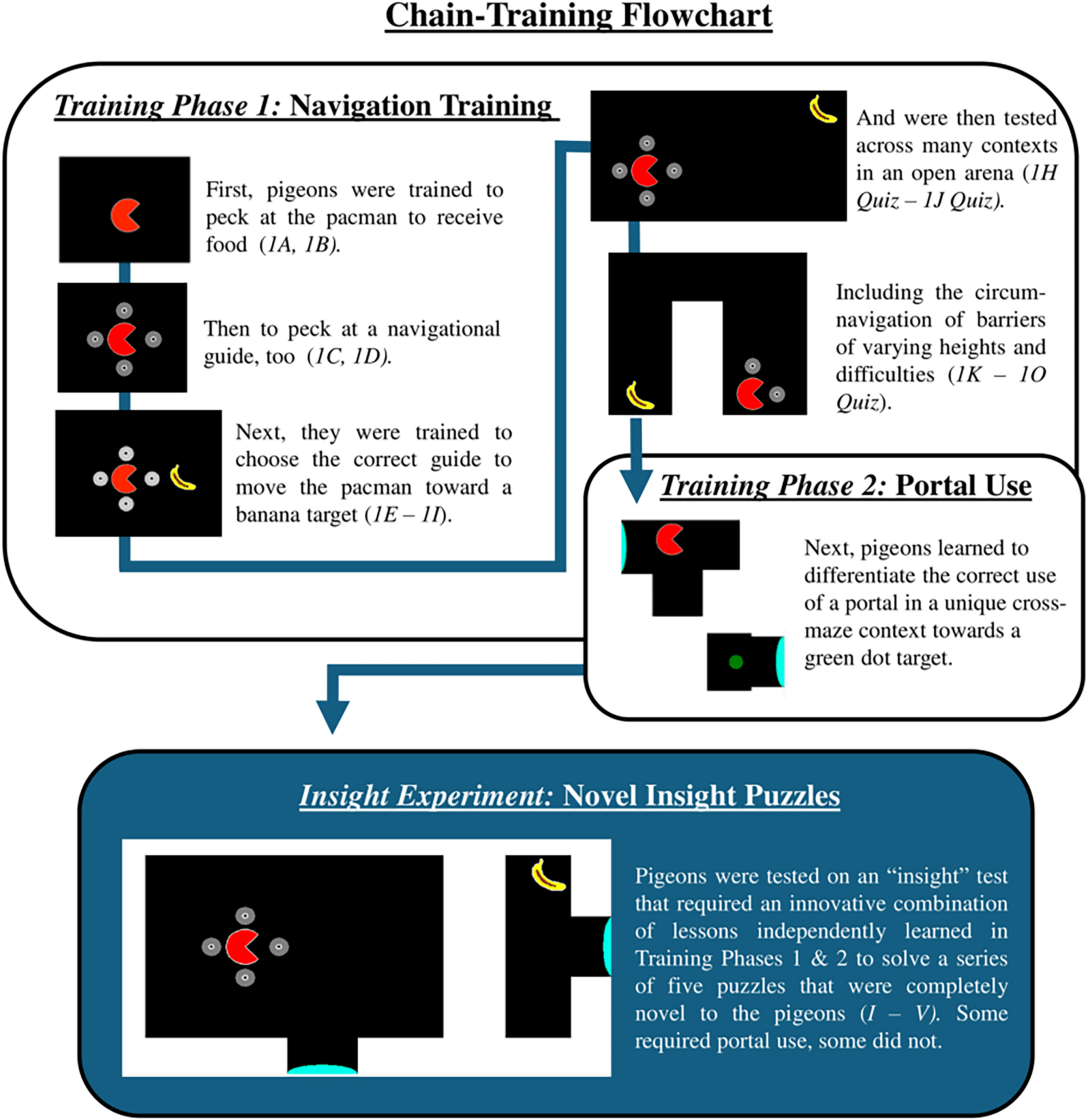
Flowchart of the behavioral‐chain procedure that was developed to train pigeons to navigate a virtual pacman avatar around a virtual environment. After basic navigation training and “tool” training in two separate environments (barriers and portals), pigeons were tested on a series of one‐shot novel puzzles that facilitated insight.

## METHOD

### Subjects

The experiment began with eight adult homing pigeons (*Columba livia*), but only three (Herriot, Wario, and Yoshi) progressed through training and reached the tests. All pigeons had previous experience with various behavioral experiments involving visual displays but were naïve concerning the current procedures and stimuli. Pigeons were individually housed in steel home cages with metal wire mesh floors in a vivarium. They were maintained between 80% and 85% of their free‐feeding weight, with free access to water and grit while in their home cages. Testing and training occurred during the light portion of the 12‐hr light–dark cycle. The procedures used in this experiment were conducted under approval and following the guidelines established by the IACUC of UCLA.

### Apparatus

Experimental manipulations were conducted in six flat‐black Plexiglas chambers (38 × 36 × 38 cm [width, depth, height]). All stimuli were presented by a computer on a color LCD monitor (NEC MultiSync LCD1550M; screen 30.5 × 21.6 cm) that was 800 × 600 (width × height) pixels. The bottom edge of the viewing window was 13 cm above the chamber floor. Pecks to the monitor were detected by an infrared touchscreen (Carroll Touch, Elotouch Systems, Fremont, CA) mounted on the front panel precisely 18 mm from the monitor screen. A custom‐built food hopper (Pololu, Robotics and Electronics, Las Vegas, NV) was located below the touchscreen at the midpoint of the front panel, its access hole flush with the floor. The hopper delivered 3.5 s of access to milo grain as food reinforcement from a hopper located behind a 4.5‐cm square food aperture centered directly below the center touchscreen. All experimental events were controlled and recorded with a personal computer operating Windows 10 OS. Stimuli were presented and data were collected using the coding language Python3 (Python Software Foundation, https://www.python.org/). The procedures were coded largely from scratch to ensure experimental control and rigorous data formation using the Tkinter (Lundh, 1999) and Numpy (Harris et al., 2020) libraries. All experimental code, analysis, and data are available via FigShare (see Supplementary Materials). Code was explicitly structured and annotated to facilitate adaptation to other labs' apparatus and encourage replication of and extensions on our procedure. It can be easily translated to screens of different dimensions and resolutions.

#### Stimuli

**FIGURE 2:**
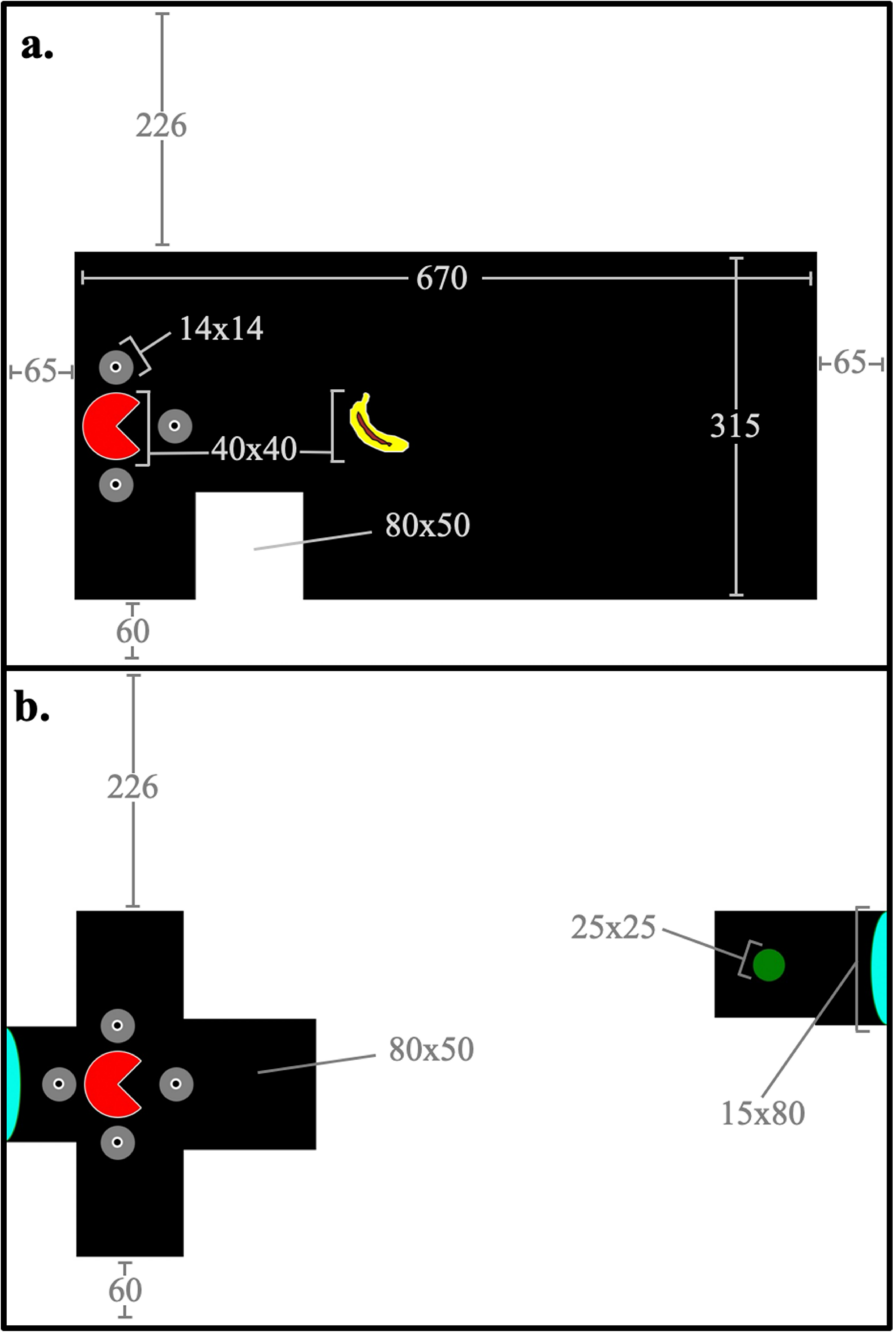
Layout and dimensions of stimuli used across training phases and the insight tests. Screenshot examples are from Training Subphase 1 K (**a**) and 2 (**b**). Provided units are in pixels.

A set of eight classes of stimuli was built to be presented to pigeons during training and experimental trials (see Figure 2 for a visual representation of stimuli). Stimulus sizes are reported in pixels, which were approximately 0.37 mm^2^ in size in our operant box. The display consisted of a black area (670 × 315 pixels) bounded by a white border. The black area served as the arena in which the pacman could be moved and stimuli could be presented. The borders consisted of four white rectangles acting as walls through which the pacman could not move. The top border was an 800 × 225‐pixel block, the bottom was 800 × 60 pixels, and the left/right borders were 65 × 600 pixels.

A pacman (40 × 40 pixels, red with a white border) icon served as the avatar, and small gray circles (14 × 14 pixels) with a white inner border and a black inner circle served as guides. A banana icon (roughly 40 × 40 pixels, banana shape in yellow with a brown stain and a white border) served as the target in Training Phase 1 and experimental trials. A small, green spot (25 × 25 pixels) served as a target in Training Phase 2. White rectangles (80 × 50 pixels) served as barriers that could be placed within the arena to restrict the movement of the pacman; multiple barriers could be connected to build complexity. A black square (80 × 80 pixels) with a turquoise semicircular base (15 × 80 pixels) served as portals, used in Training Phase 2 and experimental trials.

### Procedure

Pigeons advanced across training subphases at variable rates dependent on performance, with each subphase progressively built on the last in a chain‐like trajectory. Training was split into two phases: (1) arena navigation training, where pigeons learned to navigate a virtual pacman avatar through an open arena grid and (2) portal training, where they learned discriminative use of portals that could transport the avatar from one side of the display to another. In Training Phase 1, pigeons navigated through a large 3 × 6 grid toward a banana goal. In Training Phase 2, they traversed a constrained plus maze toward a green spot goal. After training was complete, we tested generalization of these two training phases to a novel arena task containing a series of puzzle configurations that had not been seen previously.

Given the adaptation of the procedure developed by Miyata et al. (2006) and Miyata & Fujita (2012), it was necessary to make some open and on‐the‐fly adjustments during the course of this study. Thus, aspects of training were adjusted based on subject performance and were often adapted to optimize acquisition performance. Performance was quantified using a range of variables, including response latency, pecking accuracy, correct choices, and—after sufficient training—optimality of path navigation. In a separate manuscript (Kirkman et al., 2025), we include the technical and statistical details of each stage of training. Given the focus of the current study on the insight tests after training was complete, we describe training and document criterion levels of performance at pivotal points of training (called “quizzes”). A threshold of *p* < .05 was maintained for statistical significance of test results across all phases.

Training, quizzing, and insight‐testing sessions often consisted of a variable number of trials but were always capped at a maximum of 75 reinforcers (each consisting of 3.5 s of access to the food hopper), as this was the point at which signs of satiation began to emerge in the pigeons. Therefore, sessions often ended with an unequal number of trials, particularly if some trials were not reinforced. The reinforcement rate per trial varied, depending on the specific subphase of training. Reinforcement was provided only at the end of a trial, during which all onscreen stimuli were removed and the display went black during hopper access. Stimuli were never visible on screen during any reinforcement interval. A 5‐s intertrial interval was initiated either immediately following the termination of food delivery or after the completion of an incorrect nonreinforced trial. All sessions were terminated after the delivery of 75 reinforcers or after 2 hr had elapsed, whichever occurred first. Sessions consisted of either training trials or testing trials but never both. Trial configurations, including reinforcement parameters, were identical across trial types. Advancement to the next subphase of training or testing was determined based on mixed criteria but was primarily based on improvement relative to the training baseline, response stability, and/or performance exceeding chance levels.

#### Training Phase 1

In this phase, we sought to replicate the results of Miyata et al. (2006) using a modified task. This task consisted of 19 distinct subphases (1A through 1O Quiz), which trained basic navigation of the pacman avatar toward the banana icon target within a virtual arena (Tables 1A and 1B).

**TABLE 1A:** Layout, goals, navigation types, and par (required moves) across Subphases 1A through 1F Quiz of Training Phase 1.

Subphase	Layout	Goal	Movement type	Par
1A	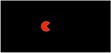	Peck Pacman at a fixed location	NA	NA
1B	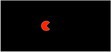	Peck Pacman at various locations	NA	NA
1C	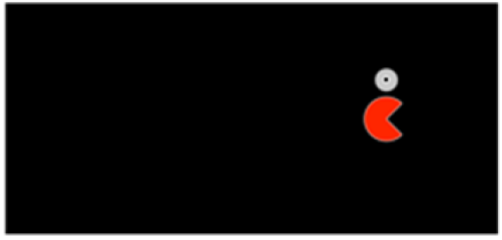	Directional guide pecking	Fixed	NA
1D	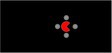	Multiple guide pecking	Differential	NA
1E	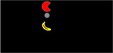	Banana as target training	Fixed	1
1F	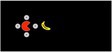	Conditional guide selection to navigate	Differential	1
1F Quiz	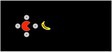	Test of guide discrimination ability	Unlimited	1

*Note*: “Movement type” refers to the number of movement possibilities the pacman had in each subphase. “Fixed” means it could only move in one direction; “differential” means it could move in multiple directions but only a few of these movements would be followed by reinforcement (i.e., reach the banana); and “unlimited” means it could move in multiple directions before it gets the banana, without restriction on the number of movements.

**TABLE 1B:** Layout, goals, navigation types, and par (required moves) across Subphases 1G through 1 J Quiz.

Subphase	Layout	Goal	Movement type	Par
1G	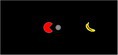	Multistep training	Fixed	2
1H	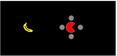	Discriminated multistep training	Differential	2
1H Quiz	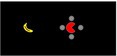	Test of multistep training	Unlimited	2
1I	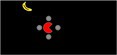	Diagonal navigation	Differential	2
1I Quiz	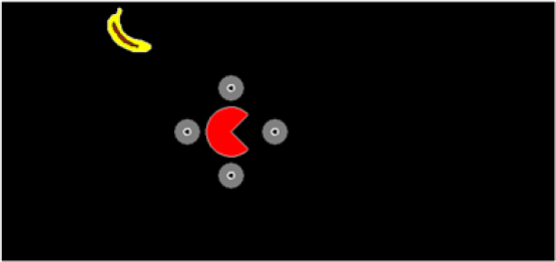	Test of diagonal navigation	Unlimited	2
1 J Quiz	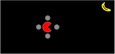	Test of full arena navigation	Unlimited	1–7

*Note*: “Movement type” refers to the number of movement possibilities the pacman had in each subphase. “Fixed” means it could only move in one direction; “differential” means it could move in multiple directions but only a few of these movements would be followed by reinforcement (i.e., reach the banana); and “unlimited” means it could move in multiple directions before it gets the banana, without restriction on the number of movements.

Pigeons were first trained to peck at the pacman when it appeared on the display in both fixed (Subphase 1A) and variable (Subphase 1B) locations within the arena. Pecks outside of the response‐sensitive boundary surrounding the pacman were documented but caused no changes within the trial. After reliable pecking was established, training on the use of directional movement cursors (“guides”) began. An initial peck on the pacman caused the presentation of one or more guides to appear at one or more of the four cardinal directions around the pacman (N, S, E, and W). A single peck on any guide caused the pacman to move a fixed distance of 120 pixels in the direction of the selected guide (hereafter called a “step”), thereby enabling pacman navigation. After the pigeons efficiently learned this two‐response chain (Subphase 1C), we changed the procedure and presented four directional guides simultaneously such that the pigeons had a choice of which direction (N, E, S, W) to move the pacman (Subphase 1D). When the pacman was near a wall, the guide in the direction of the wall was not presented. Additionally, the constraints imposed by the walls of the arena forced pigeons to vary their cursor choices (e.g., pigeons could not simply move north across more than three consecutive trials).

**FIGURE 3:**
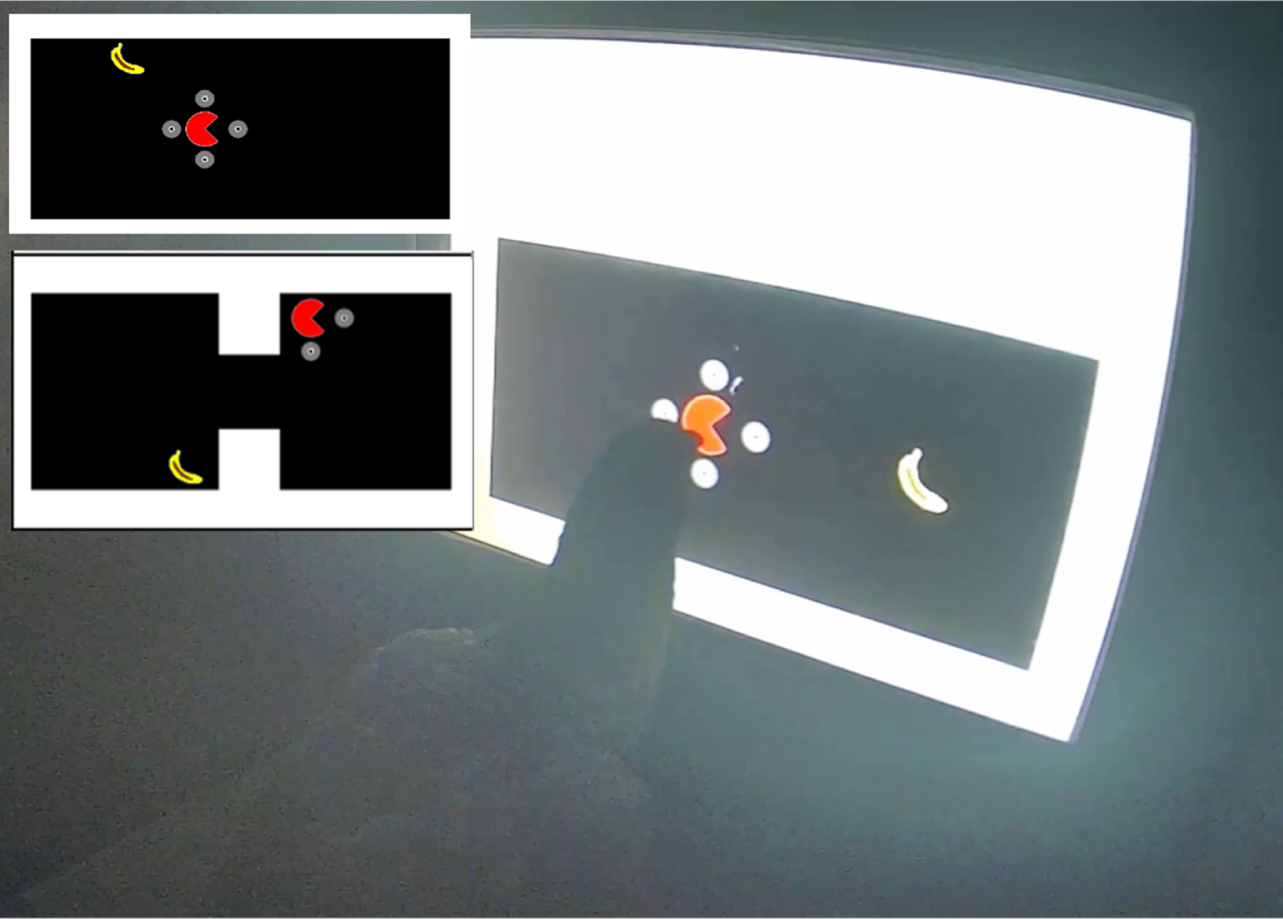
Screenshot of Herriot playing pacman in Training Phase 1 (Subphase 1H). Inset: Two examples of displays used to train pigeons to navigate a pacman to a banana on a computer screen. The upper panel contains no barriers (Subphase 1I), and the lower panel contains two barriers (Subphase 1 N).

After pigeons learned to use the guides, we trained selective directional movement of the pacman (Subphases 1E to 1H). This was accomplished through the introduction of a banana icon to serve as a target of pacman movement. A peck to the banana had no effect, but if pigeons successfully navigated the pacman in the direction of the banana (e.g., pecked a guide that appeared between the pacman and banana), the pacman would move to superimpose on top of the banana and an animation would show the pacman closing its mouth, becoming a complete filled‐in circle that appeared to a human observer as though the pacman was eating the banana. Whenever this animation was shown in this and following subphases, reinforcement was delivered. Throughout all relevant subphases of Training Phase 1, the banana appeared one (Subphases 1E, 1F, and 1F Quiz), two (Subphases 1G–1I), or a variable (Subphases 1I Quiz–1O Quiz) number of steps away from the pacman at the start of a trial. Figure 3 shows one of the pigeons (Herriot) on Subphase 1H.

At first, the banana was one step away from the pacman and only a single guide was present, located between the pacman and the banana (Subphase 1E). After the pigeons' behavior came under the control of this contingency, up to four guides appeared around the pacman (Subphase 1F). Now only a *correct* guide choice (e.g., movement toward the banana) was reinforced. If pigeons selected the incorrect guide, the pacman moved in the selected direction, paused, and then the trial ended without reinforcement.

Next, we removed the punishment constraint of requiring movement directly toward the banana in the first step such that the pacman had free range of the entire arena with an unlimited number of steps (Subphase 1F Quiz). This allowed us to assess the proclivity of pigeons to use the guides to navigate the pacman, thus “quizzing” open‐arena navigational ability. Each trial concluded with reinforcement once the pacman reached the banana. Although the pacman always started at a location one step away from the banana in this subphase (and trials *required* only a single step for reinforcement), trials could theoretically take a near‐infinite number of steps before the banana was reached. In fact, one subject took 134 steps within a single trial to reach the banana during quizzing.

The following set of training subphases (1G–1H Quiz) placed the banana two steps away from the pacman's starting position. Here, the same guide peck (e.g., north, north) was required within each trial. Similar to the previous training (Subphase 1E), only the correct navigational guide appeared at first (Subphase 1G), and all trials were reinforced. Next, up to four guides appeared around the pacman and only trials on which the pigeon made two consecutive correct guide choices were reinforced (Subphase 1H). If the pacman did not reach the banana after two steps, the trial ended without reinforcement. To test performance, pigeons were again given free range of the arena and an unlimited number of steps to reach the banana (Subphase 1H Quiz).

After pigeons demonstrated an ability to selectively navigate toward a banana target two steps away, we tested for multidirectional navigation (Phases 1I and 1I Quiz). In these novel layouts, the pacman and the banana were placed in various locations but always diagonally with respect to each other and thus required two different movements. At first, only trials in which the pigeon made two consecutive correct guide choices were reinforced (Subphase 1I); then, pigeons were allowed an unlimited number of steps (Subphase 1I Quiz). Next, we tested for generalization of learned navigation behavior by placing the pacman in one of 18 quasirandom locations within the arena on each trial and placing the banana anywhere between one and seven steps away (Subphase 1 J Quiz).

In the next stages of training, we started introducing a single barrier block that covered one space within the 3 × 6 arena. It could appear in any location that was not against the vertical walls (e.g., in vertical Columns 2–5). The barrier was visually and functionally similar to the walls surrounding the arena (i.e., impenetrable; see Table 2). The pacman began on the left adjacent side of the barrier and the banana on the right (Subphase 1 K), but the display height of the pacman, barrier, and banana could be in any of the three vertical locations. Therefore, par (the minimum number of steps to reach the banana) could vary from two to four. After performance stabilized, the left–right positions of the pacman and banana were flipped (Subphase 1 L).

**TABLE 2:** Layout and attributes of each subphase 1 K through 1O quiz of Training Phase 1.

Subphase	Layout	Goal	Barriers	Par	Pacman:banana orientation
1 K	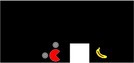	Barrier navigation	1	2–4	L:R
1 L	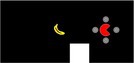	Navigation generalization	1	2–4	R:L
1 M	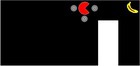	Circumnavigation	2	2–6	L:R
1 N	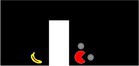	Circumnavigation generalization	2	2–6	R:L
1O Quiz	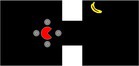	Testing	1–2	2–9	Variable

Next, a second barrier was presented in vertical alignment with the first. The barriers could be either stacked vertically or placed at the top and bottom of the arena but were always passable by circumnavigation (par thereby varied from two to six steps). We again began with the pacman consistently placed to the left of the barriers and the banana to the right (Subphase 1 M) and then flipped these positions (Subphase 1 N). Two pigeons (Bowser and Darwin) had so much trouble with this task that they were unable to meet performance criterion and were dropped, even after as many as 11 consecutive Subphase 1 L sessions (versus only 2–3 sessions to criterion by the remaining pigeons Herriot, Yoshi, and Wario).

The final subphase of arena training (1O Quiz) tested for generalization of barrier navigation to varying trial types. Trials were structured similarly to those during previous training (Subphases 1 K–1 M), using any combination of either single‐ or double‐block barriers, but now the pacman and the banana could be located anywhere within the arena (i.e., no longer always adjacent to the barrier). Therefore, par varied much more across trials and could be between two and nine steps.

#### Training Phase 2

Training Phase 2 used a novel display layout and a novel target stimulus to train the use of an abstract tool for navigation: “portals.” We took inspiration from Rodrigues and Garcia‐Mijares (2021) in which the Portal 2 video game was used to create a series of interactive puzzles to investigate the influence of extinction on insight and problem solving in humans. The three pigeons that completed Training Phase 1, Herriot, Wario, and Yoshi, participated in the second training phase.

**FIGURE 4:**
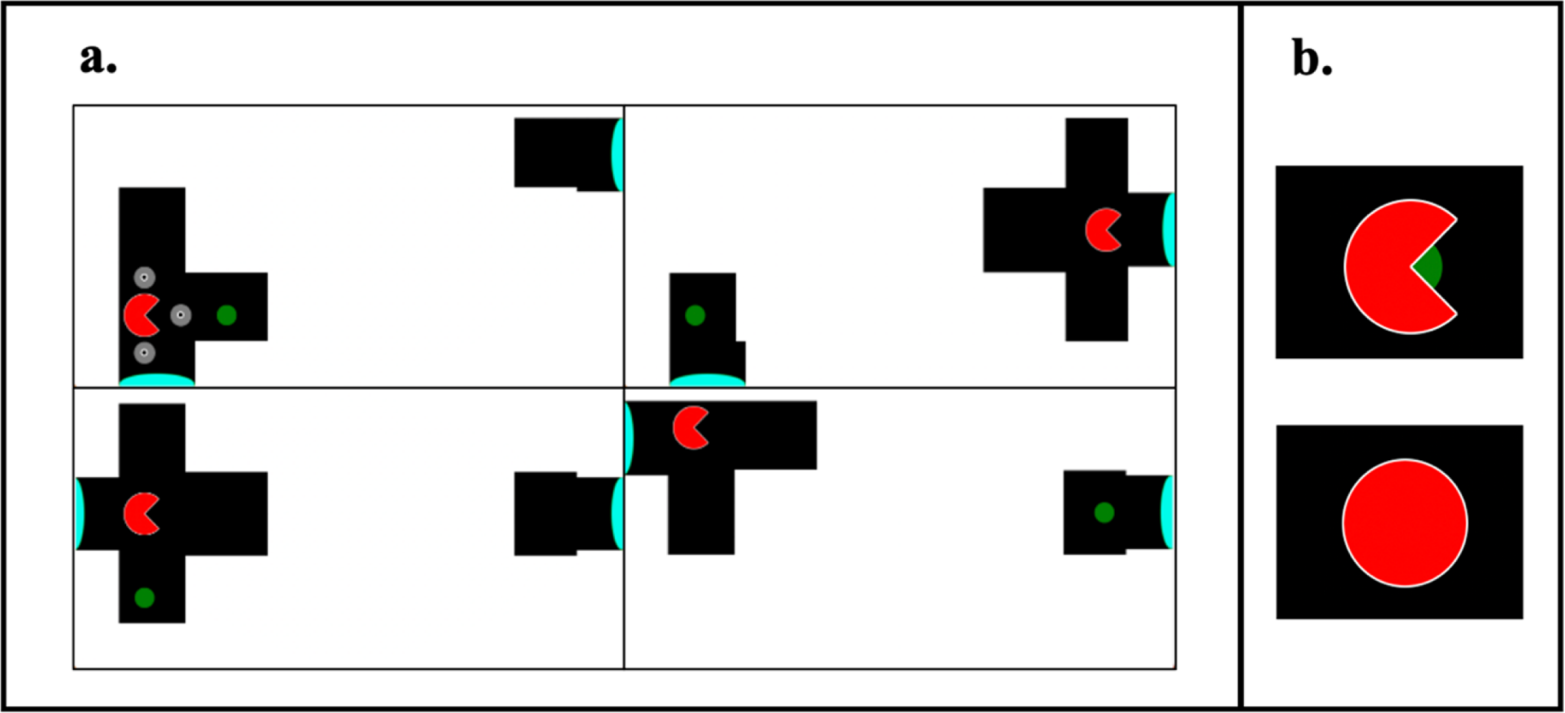
Visualization of Training Phase 2 trial layout. In Training Phase 2, the terminal‐link stimulus prior to each trial's reinforcement featured the pacman sitting “over” the green dot (**b, top**). In Training Phase 1 and the insight tests, the terminal link was instead an enclosed circle (**b, bottom**) that completely enclosed the banana (e.g., “eating” the stimulus). Both terminal‐link stimuli remained onscreen for 750 ms before reinforcement.

Training Phase 2 featured a single session type that was designed to train pigeons to navigate the pacman through a portal to reach locations in the arena that were inaccessible via direct navigation. The layout of the display was unique in that it consisted of a “plus”‐shaped navigable space on one side of the display and a separate navigable space on the other side of the display that could not be reached directly (see Figure 2b for stimulus dimensions). In Figure 4, four potential trials are shown (Panel a) with navigational guides visible in the top left quadrant. Portal use was required on about 30.3% of trials (e.g., top‐right and bottom‐right panels of Figure 4a).

If the pacman traveled in the direction of the portal (e.g., by pecking the guide in the direction of the portal), the pacman would energetically move in that direction until it was fully eclipsed by the first portal and then would reappear within the opposite portal and incrementally move out into the open space parallel to the second portal.

To train conditional use of the portal, we introduced a novel target that was used only in Training Phase 2: a green spot (cf. Epstein et al., 1984). The banana was never present in this training phase. The green spot could appear in any of the traversable spaces around the pacman—including in the space that could be reached only by navigating the pacman through the portal—and was therefore always a single step away from the pacman at the start of each trial. Thus, all Training Phase 2 trials had a par of one. On each trial, pigeons were required to move the pacman in the direction of the green spot to receive reinforcement but had no constraint on the number of steps they could make such that they could move the pacman an unlimited number of times until it reached the green spot. To further differentiate between the current green spot and previous banana goals, the pacman did not energetically “eat” the green spot once it was reached but simply sat on it for 750 ms with its mouth open and the green spot still visible (Figure 4b). On some trials (approximately 30.3% or 23 trials each session), the green spot was on the opposite side of the arena, far away from the pacman, and could be reached only by using the portals. But, on other trials (the remaining 69.7% or 55 trials), the green spot was on the same side of the arena and using the portals would inefficiently move the pacman away from the green spot, thereby delaying their access to food and requiring them to navigate back through the portal to the side with the green spot. Thus, by training pigeons to discriminate between these various types of configurations, we could establish conditional control by the function of the portal (i.e., to teleport the pacman)—that is, to use the portal when it was necessary to reach the target but not when it was unnecessary to reach the target—rather than discriminative control by its mere presence. Portals, pacman, and green spot positions were quasirandomized on each trial given the constraints above such that pigeons were presented with various configurations of the same type of task.

#### Insight tests

In Training Phase 1, pigeons acquired discriminated navigation of a pacman avatar around barriers and toward a banana target in an open virtual arena. In Training Phase 2, pigeons learned conditional use of the portals to navigate toward a green dot in a virtual cross‐type maze. The final insight tests (Puzzles I–V) served as a culmination of each of these precursor steps and procedurally combined navigation in an arena containing barriers and portals (Table 3). The basis of the test was a conceptual replication of the insight test used by Epstein et al. (1984) in which pigeons that had the appropriate types of prior training could solve the novel problem at test relatively quickly and efficiently. The goal was to replicate the insightful behavior that Epstein et al. and Köhler (1925/1959) observed and further investigate the component processes of it.

**TABLE 3:** Spatial layout, goals, par, and portal efficacy across all puzzles of the insight experiment.

Puzzle	Layout	Goal	Par	Portal usage efficient?
*I*	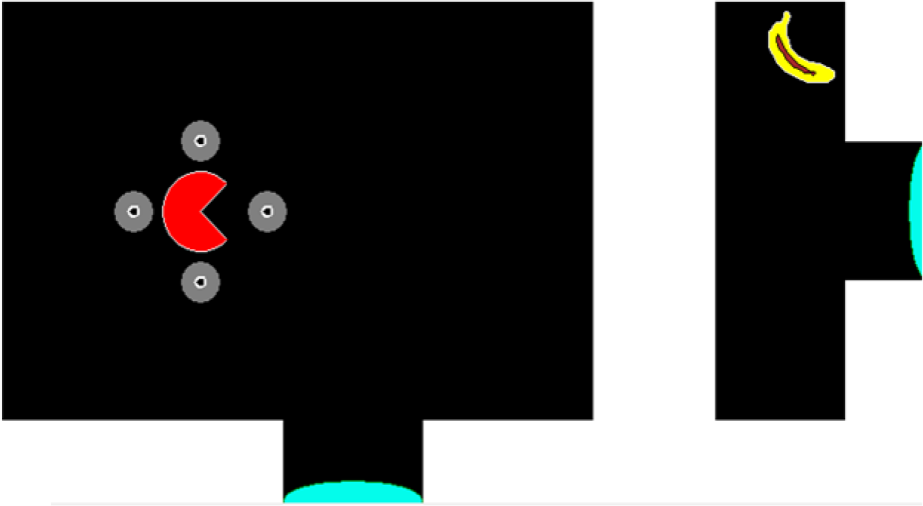	Insightful portal usage in a novel puzzle with impassable barrier	4	Yes
*II*	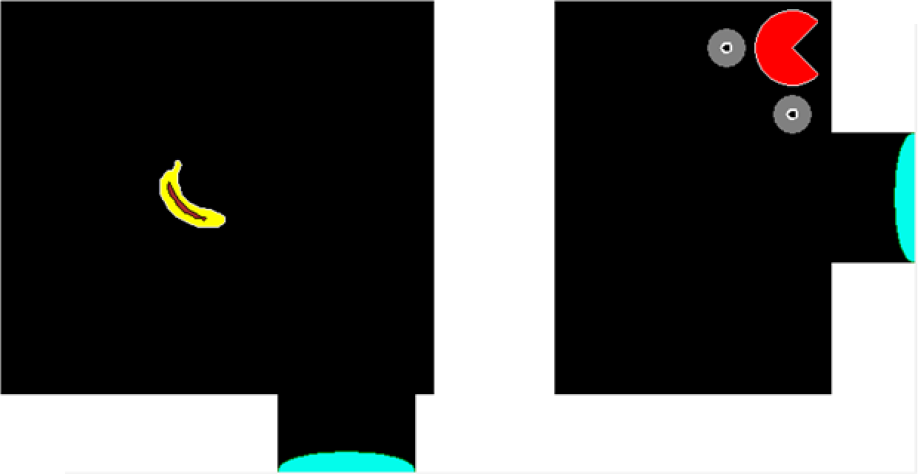	Navigation toward portal is away from banana	4	Yes
*III*	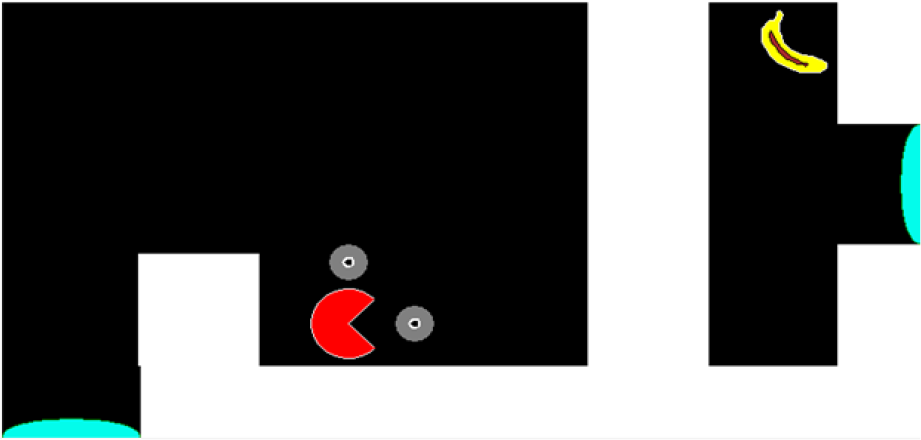	Circumnavigation around barrier to reach portal	6	Yes
*IV*	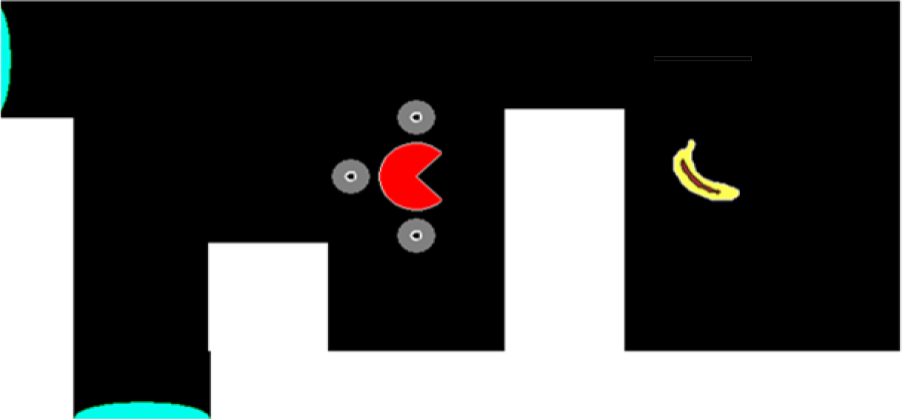	Discrimination of portal necessity (or explicit avoidance of portals	4	No
*V*	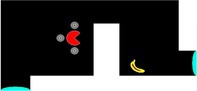	Preference for direct or portal navigation	5	Equal

Herriot, Wario, and Yoshi completed the insight tests. Across the pigeons' experimental history, the banana target had only been used during pacman navigation around visual barriers, whereas the green spot in Training Phase 2 was used to train pigeons to navigate the pacman through portals. Thus, solving the novel problems presented in the insight tests required functional transfer from the green spot to the banana, similar to the approach used by Epstein et al. (1984).

Pigeons were presented with a single test session consisting of five distinct novel displays, each with a different configuration but all with the same objective: to navigate the pacman to the banana. Each of the five puzzles was designed to assess components of behavioral processes of insight. These include, among others, trial‐and‐error learning, acquired equivalence, and autochaining (a full discussion of the various processes that may be involved in insight in this task is covered more fully in the Discussion). All five puzzles were novel to the pigeons before the commencement of the insight tests. Although each puzzle was different, they all maintained a shared logic: use of the portals to reach the banana when they were useful and avoidance of the portals when they were not.

Each puzzle was presented once sequentially to serve as the primary assessment of insight performance. Following the first run‐through with each of the five puzzles, pigeons received a second round of sequential testing in the same order. After the second round, pigeons continued to receive presentations of the various puzzles in quasirandom order for the remainder of the insight test session. Food reinforcement was delivered on each trial when the pacman reached the banana; thus, the first trial of each puzzle provides the cleanest analysis of performance on that puzzle prior to any history of reinforcement. The repeated trials provide additional data from which to explore additional questions (see below). Testing continued until 75 trials were completed or the maximum session time (100 min) had elapsed, whichever came first. Puzzles could never be repeated more than three consecutive times.

In the first three puzzles of the insight tests (Puzzles I, II, and III), the banana and the pacman were completely separated by a white barrier running continuously from the top to the bottom of the arena, thereby separating it into two unconnected spaces and blocking direct access to the banana (see Table 2). Two portals were present, one on either side of the barrier, thereby granting access to the banana. The initial three puzzles tested whether pigeons would navigate through the portal to reach the banana given a novel insurmountable barrier or would attempt to navigate directly toward the banana as they had been trained to do across Training Phases 1 and 2.

The two remaining puzzles (Puzzles IV and V) included portals but did not *require* navigation through them to efficiently reach the banana. Our objective was to observe whether pigeons would discriminate whether portals were irrelevant or relevant to reaching the banana. Puzzle IV featured irrelevant portals positioned so that their use would move the pacman farther away from the banana. Here, navigation toward the banana (and ignoring portals) would suggest an ability to discriminate between contexts in which portals were useful and those that were not (e.g., discrimination of portal's function), as demonstrated in Training Phase 2 with a green spot target and a reduced arena setting.

Puzzle V featured a problem in which navigation to the banana using portals required the same number of steps as simply circumnavigating around a barrier. This effectively allowed us to gauge the balance of stimulus control by the portals versus the barriers. From a strictly economic standpoint, the portal route took approximately 750 ms longer than the spatial‐navigation route given that a move through a portal had a longer animation than other movements; however, this difference is minimal enough that it is likely be irrelevant to the pigeons.

## RESULTS

Of the eight pigeons that began this study, only three were able to progress to the final experiment (see Table 4 for subject details). The three pigeons completed a mean of 92.3 sessions across more than 20 different training and “quiz” session types to probe translational performance on novel tasks.

**TABLE 4:** Sessions and trials (in parentheses) completed per training phase, subphase, and experiment for each pigeon.

Phase	Subphase	Subject							
		Herriot	Wario	Yoshi	Bowser	Darwin	Estelle	Athena	Mario
1	1A	6 (209)	6 (306)	3 (198)	5 (55)	6 (306)	6 (277)	6 (306)	2 (100)
	1B	6 (207)	6 (336)	3 (198)	6 (336)	4 (264)	6 (243)	6 (206)	3 (138)
	1C	10 (396)	11 (465)	10 (628)	11 (726)	11 (551)	11 (414)	11 (649)	9 (318)
	1D	5 (330)	5 (330)	5 (318)	5 (265)	5 (330)	5 (279)	5 (330)	5 (330)
	1E	6 (396)	7 (462)	7 (462)	7 (397)	7 (462)	7 (462)	7 (462)	8 (50)
	1F	4 (628)	6 (675)	6 (621)	6 (612)	6 (641)	6 (658)	6 (68)	X
	1F Quiz	6 (456)	4 (229)	4 (304)	2 (88)	2 (98)	2 (26)	2 (4)	X
	1G	6 (920)	5 (580)	5 (705)	2 (86)	2 (93)	1 (38)	X	X
	1H	3 (374)	3 (228)	3 (330)	3 (313)	9 (496)	9 (414)	X	X
	1H Quiz	2 (48)	2 (90)	2 (142)	6 (391)	12 (538)	13 (573)	X	X
	1I	10 (550)	3 (228)	5 (332)	8 (454)	4 (394)	7 (459)	X	X
	1 J Quiz	2 (152)	6 (456)	6 (367)	5 (239)	2 (152)	6 (103)	X	X
	1 K	5 (380)	1 (76)	2 (152)	5 (314)	5 (332)	18 (243)	X	X
	1 L	1 (76)	4 (162)	5 (380)	2 (109)	10 (419)	X	X	X
	1 M	2 (152)	3 (11)	2 (116)	11 (321)	2 (34)	X	X	X
	1 N	3 (185)	3 (81)	3 (161)	7 (92)	X	X	X	X
	1O Quiz	7 (264)	10 (545)	6 (339)	X	X	X	X	X
2		5 (375)	8 (608)	10 (409)	X	X	X	X	X
Insight experiment		1 (57)	1 (71)	1 (19)	X	X	X	X	X

*Note*: The letter “X” indicates that a certain pigeon did not participate in that phase or subphase.

### Training Phase 1

Pigeons began with performance just above baseline (chance level approximately 35% determined by possible pacman/guide positions in the arena), achieving a mean accuracy of 39.89% (*SD* = 6.50%) in their first session, but quickly improved to 64.57% mean accuracy (*SD* = 17.70%), which was significantly greater than chance performance by each pigeons' terminal session as assessed with a one‐sample *t* test, *t*(6) = 4.42, *p* < .01. Table S1 shows more details of the learning metrics for these subphases. Mean latencies on initial trials are consistently higher than those on final trials for most subphases. Results from regression models (general linear model and beta regression) in Table S1 also show that in most phases, pigeons performed significantly better as trials progressed.

To measure within‐trial efficiency in Subphase 1F Quiz and beyond, we constructed a metric for gauging performance: steps above par (SAP). Inspired by the golf term of par (but not exactly conforming to it), SAP represents the number of residual steps taken on a given trial after subtracting the minimum number of steps necessary to reach the banana. For example, if the banana could be reached in one step (a “par one course”) but it took a subject three steps to reach the banana, then SAP for the trial was calculated to be two steps. More efficient performance translated to a lower SAP, which was always equal to or greater than zero. To gauge unconstrained performance relative to chance, we compared subject SAP scores to performance by a random‐walk control. Our random‐walk model used an algorithm that was fed the same parameters as those for the current training subphase (i.e., arena dimensions, pacman/banana locations) and, for each trial, randomly moved a virtual pacman around the arena until the banana was reached, thereby generating a range of comparison SAP scores across the same variety of trial types that pigeons experienced. This algorithm was loosely based on a two‐dimensional random‐walk model (hereafter RWM) and served as the comparison population for our experimental pigeons. Statistical comparisons to pigeon performance featured quasirandom selection of generated RWM trials to match population sizes and used a Wilcoxon rank‐sum test (e.g., Mann–Whitney *U*) to measure differences across populations with drastically different variances (Mann & Whitney, 1947).

Of the eight pigeons, only four were able to complete the 1F Quiz subphase, as Athena and Mario were dropped after failing to reach the performance criterion. The remaining pigeons performed significantly better (SAP mean = 2.36, *SD* = 2.20) than the RWM population (SAP mean = 15.15) in their terminal Subphase 1F Quiz session, after which they were deemed ready to move on to the following training subphase.

Several pigeons showed efficient responding within only a couple of sessions during the test (Subphase 1H Quiz) that followed the discriminated multistep training (Subphase 1H). Wario, for example, showed a mean SAP of 2.11 (*SD* = 8.23) in his first session. Other pigeons took as many as 15 Subphase 1H Quiz sessions to show comparable efficiency. By their terminal session of the Subphase 1H Quiz, all remaining pigeons significantly outperformed the RWM (Table S2). Similar results were observed after training diagonal navigation (Subphase 1I), as the pigeons easily outperformed the RWM in their first session of the Subphase 1I Quiz (Table S2).

Results from the test of generalization of learned navigation (Subphase 1 J Quiz) showed that most pigeons (Bowser, Darwin, Herriot, and Wario) performed very efficiently on their first session (mean individual SAP = 5.11, 3.44, 2.16, and 1.71, respectively; individual *SD* = 6.79, 8.39, 4.12, and 5.42, respectively; see Table S2 for RWM performance). Remaining pigeons Estelle and Yoshi initially struggled to complete these novel trial configurations (mean SAP = 5.64 and 18.00, *SD* = 10.91 and 26.28, respectively), especially in trials with a higher par (GLM estimate: trial par ~ mean SAP = 0.79, *SE* = 0.24), *t*(6) = 3.32, *p* < .05. Nevertheless, by their sixth session of the Subphase 1 J Quiz, performance improved drastically and was comparable to that of the other pigeons (mean SAP = 2.22 and 0.56, *SD* = 5.00 and 1.12, respectively). Pigeons showed better performance on trial types with a lower par, as evidenced by a positive correlation between par and mean SAP (GLM estimate: trial par ~ mean SAP = 1.31, *SE* = 0.30), *t*(6) = 4.44, *p* < .01.

Pigeons showed learning in the first session encountering barriers (Subphase 1 K), with a significant decreasing trend in SAP across trials (combined GLM estimate: SAP ~ trial = −0.0175, *SE* = 0.007), *t*(6) = −2.55, *p* < .05, and trial completion time (combined GLM estimate: trial duration ~ trial = −2.071, *SE* = 0.962), *t*(6) = −2.15, *p* < .05. SAP was consistently better than the RWM control (mean RWM SAP = 39.5). One bird (Estelle) struggled with the barrier circumnavigation task, and thus she was dropped from the study after 17 sessions of Subphase 1 K.

**FIGURE 5:**
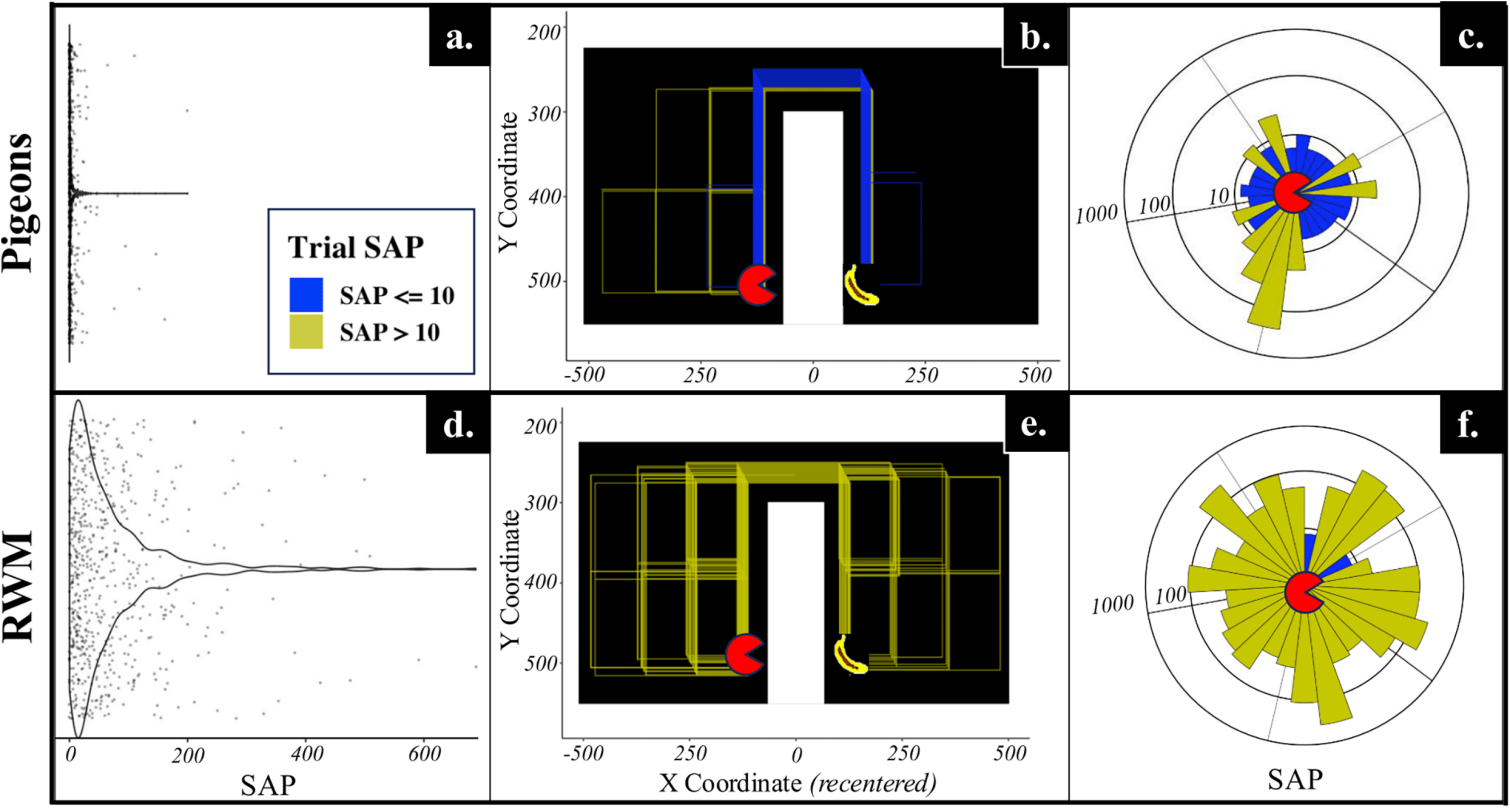
Combined pigeon performance (Top: **a**, **b**, **c**) compared with random‐walk model (RWM) control (Bottom: **d, e, f**) in Subphase 1 N. Trials with SAP scores up to and greater than 10 are differentiated by blue and yellow, respectively (**b, c, e, f**). An equal number of like trials were quasirandomly selected from the RWM control population for comparison (**e, f**). Path coordinates are centered around a central value to account for spatial variance in barrier location (**b, e**), but relative location of the pacman to the banana remain unchanged. Pigeon performance is combined across the three pigeons that participated in Phase 1 N to illustrate general differences between pigeons and RWM performance.

Figure 5 shows different comparisons between pigeons and the RWM population on Subphase 1 N (i.e., circumnavigation generalization training). The violin plots (**a**, **d**) show comparative SAP scores across the entire subphase, and the remaining plots (**b**, **c**, **e**, **f**) compare performance within a specific Subphase 1 N trial type with the largest possible par of six. (This specific trial type was encountered 27 cumulative times across all pigeons; each subject encountered it at least once.) Paths taken from start (pacman) to finish (banana) are also shown in Figure 5 (**b**, **e**), with each offset line representing one of these 27 trials. The sunburst plots in Figure 5 (**c**, **f**) display the variance of the SAP scores, wherein each ray represents one trial (of the same 27) and the length of the ray indicates the number of moves taken to reach the banana. All these comparisons also strongly indicate that pigeons outperformed the RWM population in these subphases. We observed a positive correlation between trial par and SAP (GLM estimate: trial par ~ SAP = 1.02, *SE* = 0.30), *t*(6) = 3.42, *p* < .001), meaning that the closer the pacman and banana were in the arena, the better performance became.

Results from the Subphase 1O Quiz showed that the three remaining pigeons (Herriot, Wario, and Yoshi) performed significantly better than the RWM control in their first test session and improved even further by their terminal testing session (see Table S3). By the completion of Training Phase 1, these three pigeons were capable of efficiently navigating a pacman avatar around barriers to reach a target in a two‐dimensional maze assay, replicating the findings of Miyata and Fujita (2012). These pigeons showed a nonrandom directedness of navigation behavior, as they drastically outperformed the RWM at every opportunity.

### Training Phase 2

Mean SAP systematically decreased across five to nine sessions during Training Phase 2 for all pigeons (see Table S4). Pigeons showed a higher proportion of correct choices—using the portal when required and avoiding it when not—indicating they learned to use it only when needed. Performance on the three terminal sessions of Training Phase 2 was significantly above chance (30.33% given the proportion of four‐option “cross” versus three‐option “T” mazes in this training), as pigeons navigated correctly in their first choice on 60.80% of trials, *t*(8) = 5.34, *p* < .001.

### Insight tests

Three pigeons progressed to the insight tests. Herriot, Wario, and Yoshi completed 57, 71, and 19 total trials, respectively.

#### Initial trials

As in the previous phases, performance was assessed via SAP and trial latency, where lower values translated to more efficient performance. In the insight tests, we also compared interresponse intervals and “missteps.” A misstep was any movement away from the most efficient route to the banana. Missteps were positively correlated with SAP scores but captured explicit moves in the wrong direction as, oftentimes, SAP increased in units of two or more increments, as pigeons backtracked following a mistake.

Puzzle I served as an initial opportunity to navigate using the portal in this novel context. For the first time, pigeons were required to navigate the pacman in the direction opposite to the banana after the initial step in the direction of the banana. That is, once the first step to the east was made, the most efficient path to the banana was to move south, away from the banana and toward the portal to bypass the barrier. On the very first trial, all pigeons showed short latencies (mean trial latency = 34.41 s; mean interresponse interval [IRI] = 0.85 s) and uniform response patterns (see Table S1 for individual data), showing no significant linear change in IRI over the course of the trial (Herriot, Wario, and Yoshi GLM estimate: IRI ~ response number = −0.001, 0.007, and − 0.007, respectively; *p*s = .989, .522, and .409). In fact, all pigeons made their first response within 0.8 s of trial onset and made a directional movement choice within 3.1 s. This initial behavior contrasts markedly with the qualitative findings of Epstein et al. (1984) and Köhler (1925/1959) that described subjects pausing before responding on a novel “insight” trial. Pigeons also showed efficient navigation performance (mean missteps = 2.33; mean SAP = 4.67; see also Table S1 for individual data).

**FIGURE 6:**
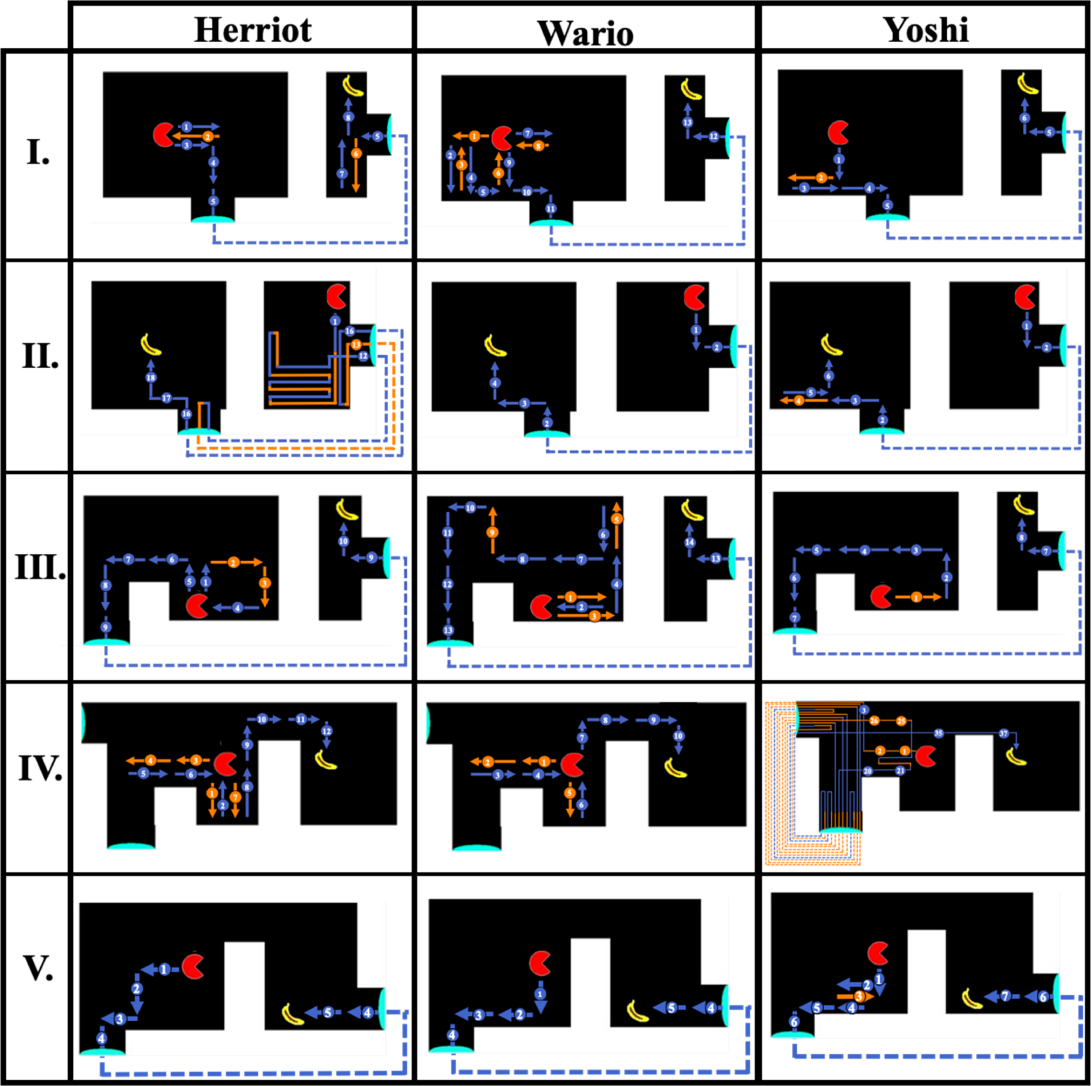
First‐trial paths of Puzzles I–V for each subject. Arrows indicate movement direction. Missteps (e.g., movements away from the most ideal path) are represented in orange; step count is numbered. Dashed lines represent portal paths.

Figure 6 shows pathways on the first trial for each pigeon. Interestingly, initial missteps on the first trial of Puzzle I were primarily in a western direction (1/2, 2/4, and 1/1 missteps for Herriot, Wario, and Yoshi, respectively), which was away from both the banana goal and portal. Once through the portal, Yoshi and Wario traveled directly to the banana but Herriot took one misstep south before correcting and navigating directly to the banana. Overall, pigeons showed immediate transfer of the spatial navigation techniques used in prior experiments on this novel test configuration.

On the second trial (Puzzle II), we reversed the display locations of the pacman and the banana and slightly shifted the location of the impassable barrier. Our objective was to determine whether pigeons would show a performance similar to that in Puzzle I when the spatial locations of the pacman and banana at the start of the trial were reversed. In their first encounter, Herriot, Wario, and Yoshi performed well (mean missteps = 2.67; mean SAP = 5.33; mean trial latency = 49.56 s; mean IRI = 0.98; see Table S1 for individual data). The performances by Wario and Yoshi largely mirrored those in Puzzle I, with near‐perfect performance (Figure 6 II shows the pigeons' pathways in this first Puzzle II trial) but differed in temporal patterns: Wario was able to speed through the trial in only 11.10 s, whereas Yoshi took over twice that time (24.01 s) to make the first move, despite pecking at the screen within 1 s of the trial starting and thus showing attention to the task. Yoshi's initial pause *was* more indicative of observations made by Epstein and Köhler. The third subject, Herriot, showed a similar pause (15.42 s) prior to making the first step.

As Figure 6 II shows, instead of navigating directly to the portal, however, Herriot navigated to the southwest corner of the accessible zone. Once the corner was reached and Herriot could not continue to navigate farther south or west due to border constraints, she moved the pacman back west, then back east to the corner, and then repeated the west–east sequence once more before moving the pacman in a north–south sequence. This apparent attraction to the southwest corner could be a function of stimulus control by the banana, as the corner was part way, in terms of display locations, between the banana and the portal. After 12 moves and 45.24 s, Herriot finally navigated away from the corner and through the portal (thus “switching” from joint stimulus control by the banana and portal, to stimulus control by just the portal) but then navigated right back through the portal and to the starting section after only 6.57 s! After a 15.93‐s north–south sequence of steps, Herriot navigated through the portal again and then directly to the banana, thus receiving reinforcement and concluding that trial. This behavior may be emblematic of a balance between discriminative control by the portals versus the banana within each trial, which continued to emerge in subsequent tests as we describe below.

Puzzle III required pigeons both to explicitly navigate the pacman away from the banana and to circumnavigate a secondary barrier to reach the portal. The introduction of a second barrier made the task slightly more difficult, as portal access now required additional circumnavigation of the barrier, and first‐trial performance mirrored this (mean missteps = 2; mean SAP = 4.67; mean trial latency = 52.59 s; mean IRI = 0.87 s; see Table S1 for individual data). Herriot and Yoshi first navigated east (a step away from the relevant portal and toward the inaccessible banana) before correcting their path, but Wario navigated all the way to the northeast corner closest to the banana before correcting and navigating toward the portal (reaching the banana soon after). The first step in Puzzle III was incorrect and east toward the banana for all pigeons, suggesting that the small barrier separating the portal could have interfered with stimulus control by the portal on that trial (Figure 6 III shows the pigeons' pathways on this first Puzzle III trial). In particular, Wario—who had perfect performance on the prior Puzzle II trial (SAP = 0)—struggled in differentiating the necessity of the portal in the first trial of Puzzle III. Pigeons' performance on the first trial of Puzzle III established an ability to navigate away from the banana target, around a barrier, and through a portal to reach the banana target but also demonstrated a factor that may influence the balance between stimulus control by the portal versus by the banana. This performance is reminiscent of the pigeons in Epstein et al. (1984) that initially tried to peck the banana before giving up and turning their attention toward the moveable box.

In sum, efficient navigation on the first three puzzles of the insight test showed strong, immediate (or rapid) transfer of spatial navigation, including around barriers and through portals, that had been acquired during previous phases of training. Although all birds showed some inefficient navigation toward the inaccessible banana, most trials featured direct navigation through the portal and to the banana.

On the first Puzzle IV trial, two of three pigeons (Herriot and Wario) successfully navigated directly to the banana without the use of the portals (see Table S1). Interestingly, both pigeons initially took two missteps toward the western portal before correctly reversing back toward the eastern banana (Figure 6 IV shows the pigeons' pathways on this first Puzzle IV trial). Both birds also moved south once instead of north before immediately correcting their course. The third pigeon, Yoshi, did not show such discrimination on the first trial. Instead, he navigated directly to the closest portal and proceeded to loop through the portals repeatedly over the next 4.5 min before finally navigating toward the banana and concluding the trial. This was the poorest first‐trial performance across all of those on the insight tests (see also Table S1). Figure 6 IV also shows that Yoshi did not repeatedly navigate toward a single portal but varied entrance/exit through both portals from multiple directions. Thus, Yoshi failed to show on this puzzle the conditional discrimination performance he had acquired by the end of Phase 2 training. Nineteen moves into this first Puzzle IV trial, Yoshi changed tactics and made three consecutive steps toward the banana (north, east, east) as if showing stimulus control by the banana before, again, falling under stimulus control by the portal (west, west, west). After 11 total portal usages, Yoshi successfully navigated away from the portals and to the banana to conclude this first Puzzle IV trial.

On the second Puzzle IV trial, however, Yoshi perfectly navigated to the banana without using an irrelevant portal (SAP = 0; trial completion = 47.74 s; mean IRI = 1.02 s). These first‐trial performances suggest that Herriot and Wario were immediately able to differentiate the function of the portal on the first trial of Puzzle IV, whereas Yoshi required some experience to learn the function (e.g., trial‐and‐error learning) or to extinguish the strong control of the portals until the control by the banana emerged. On the second trial of Puzzle IV, however, Herriot and Wario demonstrated strong attraction to the portals, similar to Yoshi's first‐trial performance. Both birds that had successfully avoided the portal on the first trial of Puzzle IV failed to do so on the second iteration of that puzzle and became temporarily stuck in the portal loop (SAP = 19. 28; trial latency = 59.93, 112.10 s; mean IRI = 0.58, 0.98 s; for Herriot and Wario, respectively). See the following section for more information on repeated Puzzle IV trials.

On the first iteration of Puzzle V, all three pigeons chose to use the portals (see Figure 6 V for the pigeons' pathways) and ended the trial with near‐perfect performances (mean missteps = 0.33; mean SAP = 0.67; mean trial latency = 29.83 s; mean IRI = 0.83 s; see Table S1 for individual data).

#### Subsequent trials

After initial presentation and completion of all five insight test puzzles, pigeons were presented with the same puzzles again to determine any within‐session changes or fluctuations of behavior, including learning through reinforcement. One remarkable feature of insight is the directedness and abruptness of behavior when solving the problem (i.e., low or complete absence of trial‐and‐error types of behavior; Epstein, 1985a; Shettleworth, 2012). Therefore, to better differentiate insight from trial‐and‐error performance, we compared first‐trial and sessionwide performance across the five unique puzzles by analyzing SAP, spatial/directional patterns, trial completion time, and IRIs. If pigeons showed significant improvement over the course of the test session, it would be evidence against insight, as they would be learning through repeated reinforced trials rather than demonstrating a direct and abrupt “insight” solution. In some prior phases, pigeons showed significant learning curves within the first sessions of a novel arena layout (like the introduction of barriers in Training Phase 1, Subphase1 K), as evidenced by decreasing linear trends in SAP and trial duration. Performance was also compared with that from both the RWM algorithm control—which all pigeons vastly outperformed, similar to previous phases—and performance in prior phases to better determine whether observed behavior could be categorized as “insight” performance similar to what has previously been observed by Epstein et al. and Köhler (1925/1959). For example, insight would be better supported if initial performance (especially via SAP) on the insight tests was similar to that in comparable trials during the three terminal sessions of Training Phase 1 (1 N Quiz). Trials with identical par were selectively compared. Similarly efficient performance would reflect insight as observed by Epstein et al. (1984) and Köhler, whereas an incremental or sharp decrease in SAP across trials of each puzzle type at test would be more emblematic of simple trial‐and‐error learning.

Across repetitions of Puzzle I, performance fluctuated but pigeons did not show significant improvements in any measures (SAP, trial latency, or IRI; see Table 5). In other words, performance did not improve across trials for this puzzle. Reiterations of Puzzle II trials also showed no significant change in SAP across trials. Nevertheless, there were significant decreases in trial latency and IRI over the course of the session (Table 5; see additional reference to these effects at the conclusion of this section). Performance on repeated Puzzle III trials showed a sustained level of performance, with no significant positive trends in SAP or IRI over the course of the session. A decreasing trend in Puzzle III trial latency across trials, however, was observed (Table 5).

**TABLE 5:** Summary of results (quantitative measures) for subsequent trials of the insight experiment.

Trial puzzle		SAP (absolute data)		SAP ~ Trial results			Trial latency (absolute data) (s)		Latency ~ Trial results			IRI (absolute data) (s)		IRI ~ Trial		
*Stat*.		*Mean*	*SD*	*GLM estimate*	*SE*	*t*	*Mean*	*SD*	*GLM estimate*	*SE*	*t*	*Mean*	*SD*	*GLM estimate*	*SE*	*t*
*I*		8.13	8.24	−0.34	0.42	0–.81	82.87	117.37	−5.161	5.95	−.87	1.08	0.41	0.001	0.03	0.21
*II*		3.69	3.14	−0.19	0.12	−1.50	34.93	15.54	−1.81*	0.55	−3.33	0.72	.10	−0.02*	0.01	−2.23
*III*		3.77	4.83	−0.35	0.19	−1.83	45.48	26.76	−2.22*	1.05	−2.12	0.98	.15	0.02	0.01	1.67
*IV*	No portal	49.83	7.55	0.55	1.80	0.30	336.53	113.51	0–.68	12.9	−0.05	1.18	0.50	−0.02	0.03	−.56
	Using Portal	4.35	4.16				45.79	12.99				0.77	0.14			
*V*		1.97	3.55	−0.27*	0.13	−2.05	30.88	18.01	−1.37	0.68	−2.03	0.89	0.24	−0.11	0.03	−.44

* *p* < .01.

Puzzle IV was the only puzzle in which the portals were explicitly irrelevant in navigating to the banana. Including the first two trial iterations in which all pigeons either successfully avoided portals (SAP = 8, 6, and 0, respectively) or got stuck in a portal loop (SAP = 19, 28, and 34, respectively), the three birds were able to discriminate portal use (i.e., did not access the portal) on just 75.61% of trials (7/10, 9/11, 6/8, respectively). This imperfect discrimination may have occurred because pigeons were repeatedly exposed to each of the five types of trials across the session, three in which portal use was necessary to reach the banana and receive reinforcement. Thus, the portals may have acquired value through conditional reinforcement and thus successfully competed with direct navigation around the barrier on trials of Puzzle IV. Puzzle IV may have become more difficult to solve as testing continued, given the increase in attractiveness of the portals on other puzzles. It is therefore difficult to determine whether these differences in pigeons' performances were a function of conditional discrimination or simply an artifact of frequency of reinforcement following use of the portals during testing. Despite the occasional demonstration of inefficient portal use on Puzzle IV, we saw no significant trend over the course of the session in all measures (Table 5).

Overall, Puzzle IV trials produced the worst performance of the five puzzles and trials in which the portal was used resulted in longer trials than those without portal use (Table 5). By the end of the session, Yoshi, the bird who originally struggled with distinguishing portal necessity on the first Puzzle IV trial, actually had better mean performance (mean SAP = 9.5; trial latency = 106.17 s) than Herriot and Wario (mean SAP = 22.5 and 13.36; trial latency = 111.50 and 114.58 s, respectively).

The final puzzle (Puzzle V) featured a spatial layout in which the pacman and the banana were equidistant via portal or direct barrier circumnavigation routes, that is, par = 5 for each route. During Puzzle V trials, all three pigeons preferred portal use (79.61%; 8/10, 10/17, 2/2, respectively) but Herriot and Wario showed flexibility in solving the puzzle, as demonstrated by direct navigation around the barrier on some trials. This flexibility may be a function of fluctuations in the strength of stimulus control by the portal versus the banana across the session or merely fluctuations in the starting attentional state of the pigeon at trial onset (that is, if the pigeon happened to be looking at the portal when the trial started, it may have been more likely to navigate to the portal).

Sessionwide performance across Puzzle V trials with portal use (mean SAP = 2.50, *SD* = 1.15) did not differ, Welch's *t*(12.98) = 0.97, *p* = .349) from trials without portal use (mean SAP = 2.46, *SD* = 2.83). Yoshi used the portal for every Puzzle V trial—which may explain his first‐trial Puzzle IV performance—but he also received only two Puzzle V trials across the session due to quasirandom trial selection. Unlike the previous four puzzles, Puzzle V performance improved across iterations, with significant linear decreases in SAP but no significant trends in trial latency and IRI (Table 5). In other words, pigeons significantly improved spatial and temporal performance across Puzzle V over the course of the session, which is likely evidence of some trial‐by‐error learning. A similar phenomenon can be observed in human decision making (Chen & Risen, 2010; Milosavljevic et al., 2010; Ratcliff & McKoon, 2008). For example, the first time one encounters a fork in the road, one may spend some time deliberating about which path to take. Once a choice is made, however, future returns to that same fork may result in faster decisions (Milosavljevic et al., 2010; Ratcliff & McKoon, 2008). In such cases, the faster response may not necessarily reflect trial‐and‐error learning, especially if both forks are reinforcing; rather, it could be due to the resolution of a concurrent reinforcing situation (i.e., choice under conflict situation). Once a choice is made, the conflict is reduced on subsequent encounters with the choice point (Chen & Risen, 2010; Ratcliff & McKoon, 2008).

No significant difference was found between SAP scores from each of the five puzzles and trial performance from similar trials in Training Phase 1 (see Table 6), suggesting that the level of performance pigeons exhibited by the end of barrier training was maintained in the novel insight trials.

**TABLE 6:** Results of insight experiment SAP comparison with trials from the terminal session of Training Phase 1.

Insight Puzzle	Comparison with terminal session of Phase 1 with identical par		
	*Welch's t test*	*df*	*p*
*I*	−0.82	70.94	.42
*II*	0.74	54.64	.46
*III*	0.09	71.79	.93
*IV*	−1.86	34.38	.07
*V*	0.97	76.97	.33

*Note*: Because insight puzzles differed in par, only the most similar trials from Training Phase 1 (1O Quiz) with identical pars were selected.

## DISCUSSION

We developed a modified virtual navigation procedure that replicated the one developed by Miyata et al. (2006) and Miyata and Fujita (2012) that served as a conceptual replication of the banana‐and‐box insight task developed by Epstein et al. (1984). Our replication used a virtual video‐game environment for pigeons. In Training Phase 1, six of eight pigeons successfully learned to navigate a pacman avatar on the touchscreen display to reach a banana icon serving as a target. Only three pigeons, however, learned to navigate the pacman around visual barriers. In Training Phase 2, all three pigeons that completed Training Phase 1 successfully learned to navigate the pacman through portals to reach a green spot that served as a target stimulus. Finally, on a series of insight tests, these three pigeons showed efficient navigation to reach the banana in novel displays that combined barriers and portals and in novel configurations.

Performance on Puzzles I, II, and III of the insight tests provides a conceptual replication of the insight performance demonstrated by pigeons in the banana‐and‐box task used by Epstein et al. (1984), which itself was a conceptual replication of Köhler's (1925/1959) study of insight in chimpanzees. We propose that performance on these tests indicated insight similar to that indicated by Epstein's and Köhler's demonstrations, as subjects rapidly produced the solution as a single, sudden, and ordered behavioral chain, without apparent hesitation or gradual approximation to the solution. Performance on Puzzles IV and V revealed a competition for stimulus control between the banana and the portals. Performance on Puzzle V also demonstrated that pigeons' behavior was flexible and improved after an initial hesitation when they first encountered a choice between two equally effective navigation options.

Thus, we successfully replicated the performance that pigeons demonstrate in real‐world versions of the banana‐and‐box insight task, such as that reported by Cook and Fowler (2014), Epstein et al. (1984), Epstein (1985b, 1987), Luciano (1991), and Neves Filho et al. (2021). What psychological processes might underlie performance on these insight tests? Table 7 provides a partial list of psychological processes that may be involved in the banana‐and‐box insight test and that could also underlie other performances on these insight tests. Although authors like Skinner (1984) and Epstein (1985a) emphasize bottom‐up aspects of insight (light shading), authors like Köhler (1925/1959) emphasize top‐down influences (dark shading). There was evidence for most of these component processes in our study. For example, on Puzzles I, II, and III, pigeons showed immediate transfer of the use of the portal to navigate the pacman to the banana after having learned to use the portal to navigate the pacman to the green spot. This suggests that functional generalization was involved. Although the term functional generalization was not clearly articulated by Epstein et al. (1984) and was just mentioned as the effect of common reinforcing history (Epstein, 1985b), in discussing Epstein's experiments, Shettleworth (2012) used the term mediated generalization. “Mediated generalization” (also known as secondary generalization) was first introduced by Hull (1939) and further explored in later studies (e.g., Urcuioli et al., 1989; Wasserman et al., 1992). For instance, Urcuioli et al. (1989) used a many‐to‐one conditional matching procedure in which different sample stimuli were mapped onto the same comparison response. Later, by reassigning reinforcement contingencies for only a subset of the original stimuli, they demonstrated that untrained sample–comparison relations could emerge, suggesting the formation of functional equivalence classes. These results support the idea that mediated generalization is a basic learning mechanism that guides novel choices based on prior associative rules.

**TABLE 7:** Behavioral and cognitive processes that could play a role during insight.

Behavioral/Cognitive process	Description	Proponents	Example
Trial‐and‐error Learning	Behavior shaped by consequences, leading to repeated successful actions	Thorndike (1898), Skinner (1984)	Pigeon learns to peck at a key for food
Primary Generalization	Responding to stimuli similar to the original conditional stimulus	Pavlov (1932), Thorndike (1898), Guttman & Kalish (1956)	Pigeon reacts to objects resembling trained stimuli
Secondary Generalization	Extension of learned behavior to new, functionally equivalent situations	Hull (1939), Urcuioli (1989; 2006)	Pigeon applies learned response to a new but similar task
Autochaining	Development of a sequence of behaviors where the end of one triggers the start of the next	Epstein (1985a, 1985b)	Pigeon combines stepping on a box and pecking to reach a goal
Discrimination	Distinguishing relevant from irrelevant features in a problem	Spence (1936), Pavlov (1932)	Pigeon ignores distractors to focus on a goal‐related object
Conditioned Reinforcement	Environmental stimuli and cues that acquire secondary conditioning value through association with primary reward	Skinner (1984)	Portal stimulus becomes an attractor due to its history of being paired with reinforcement
Behavioral Variability	Variation in behavior based on extinction/nonreinforcement; exploratory behavior	Blaisdell (2009; 2016), Roberts, Neuringer	Following the extinction of learned responses, the pigeon's behavior increasingly varies, leading to alternative solutions
Mental manipulation	Internal manipulation of problem elements or stimuli to arrive at a solution	Köhler (1925)	Chimp stacks multiple boxes to reach a banana, which may demonstrate internal planning

*Note*: Shading indicates the location of each process on the continuum of bottom‐up (no shading) to top‐down (dark shading) aspects. Light shading indicates processes that could include both bottom‐up and top‐down aspects.

In our task, pigeons received the same reinforcement for navigating the pacman to the banana and to the green spot. Thus, the banana and green spot could enter into the same functional equivalence class. As a result, any behavior pigeons had learned to do in the presence of the green spot as a target, such as navigating the pacman through the portal, could immediately transfer to the banana, which is what was observed at test. Equivalence learning could also explain the immediate transfer of pushing the box to the banana on the insight tests by Epstein and others after having been trained to push the box toward a green spot on the wall of the chamber.

Autochaining, which had been demonstrated by Epstein (1985b) and others in the physical version of the banana‐and‐box task, was also observed on Puzzles I, II, and III in our pacman navigation task. After pigeons had navigated the pacman through the portal, the pacman now being in close spatial contiguity to the banana directly elicited the type of behavior trained during Experiment 1, navigation directly to the banana. Likewise for Puzzle III, pigeons demonstrated that they could first navigate the pacman around a barrier (as in Training Phase 1) to reach a portal (as in Training Phase 2), followed by completion of the trial by navigating directly to the banana (as in Training Phase 1). Thus, the novel combination of different trained behaviors emerged spontaneously at test and followed a sequence based on the stimulus situation at each stage of the test trial that matched a prior learning stage most closely, thereby demonstrating autochaining.

Discrimination learning and occasional errors in discrimination were also observed in the test performances of our pigeons. For example, although pigeons had learned in Training Phase 2 to successfully discriminate trials where they should versus should not navigate through the portal, some of the birds failed to show such discrimination on some of the test trials. Specifically, Yoshi was caught in a “portal loop” on the first trial of Puzzle IV, and Herriot and Wario were similarly caught by the portals on a subsequent trial of Puzzle IV. This suggests the process of conditioned reinforcement. During Training Phase 2, despite showing discrimination of when to use and when not to use the portal, the portal itself is a visual stimulus that may have acquired control of behavior due to its proximity to food reinforcement and thus may have acquired properties as a discriminative stimulus. As a result, on Puzzle IV, any discriminative properties of the portal could explain how the pigeons occasionally got caught in a “portal loop.”

Nevertheless, once caught in the “portal loop” on a Puzzle IV trial, the responses of that pigeon no longer showed similar control by the portal on subsequent Puzzle IV trials. This could be due to extinction of stimulus control by the portal and the resurgence of other trained behaviors as well as an increase in behavioral variability (Blaisdell et al., 2016). Despite extinction of stimulus control by the portal, pigeons continued to use the portal on other trials when it was necessary to complete the task (e.g., trials of Puzzles I, II, and III). Just as a stimulus can retain its function as a discriminative stimulus even after its conditioned stimulus function is extinguished (Rescorla, 1986), the portal in our study also maintained its function despite extinction.

One process we have not specifically mentioned yet, often discussed in the insight or problem‐solving literature, is mental simulation. Despite the disadvantages and controversy of adopting cognitive explanations on insight (see Epstein, 1985a; Skinner, 1984), it is undeniable that insight and problem‐solving research (especially in the recent human literature, Davis, 1966; Holth, 2008) has been adopting mental simulation and other cognitive concepts as explanatory concepts to expand the scientific investigation of these phenomena (e.g., Galinsky & Moskowitz, 2000; Murray & Byrne, 2013). For example, Kounios and Beeman (2014, p. 74) define insight as “any sudden comprehension, realization, or problem solution that involves a reorganization of the elements of a person's *mental representation* of a stimulus, situation, or event to yield a nonobvious or nondominant interpretation” (italics added). Although there is much debate around whether mental representations and simulation have causal roles in explaining animal behavior, with some advocating for the usefulness of adopting this cognitive framework (e.g., Blaisdell, 2009, 2019; Neiworth & Rilling, 1987; Wessells, 1982) and others pointing to its limitations (e.g., Dwyer & Burgess, 2011; Hayes & Brownstein, 1986; Skinner, 1984), it is important to mention how the insight literature has been growing in diverse philosophical and scientific directions and highlight how our work can also contribute to this expansion as an innovative method. In addition, we also point out that there is no evidence in our current task that pigeons engaged in such mental operations. Therefore, we will not comment further about this possibility but only mention that our method could be further improved to facilitate investigating these questions.

A second goal of this study was to develop a virtual task in which to study insight. The successful insight performance of three pigeons serves as a proof of concept that achieved this goal. The virtual procedure was developed using Python software, a programming language that is commonly used in behavioral research. This development also allowed us to quantify behavior in the task. Because each movement of the pacman was discrete and trackable, we could measure each movement as a discrete step. Because there was always a minimum number of steps by which the pigeon could navigate the pacman to the target, we could set a par for each course (borrowed loosely from its use in golf). Efficiency in navigation was quantified as SAP. Missteps could also be calculated and thus provide the number of errors a pigeon made on any given trial. This allowed us to directly compare behavior against an optimal level (par), a random walk model, and the pigeons' own performance across trials and sessions as well as across pigeons.

One major concern in this study was the precision of pigeons' pecks to the touchscreen display. Precision of peck responses on touchscreens has been previously reported as an experimental limitation (Seitz et al., 2021; Toegel et al., 2021). The small physical size of our digital stimuli, particularly the navigational guides, resulted in wide variability of spatial pecking, including many missed pecks. Even after our three final pigeons had successfully completed thousands of trials by the end of Training Phase 2, pecking accuracy on guides remained quite variable (see the Supplementary Material for details). Future iterations of this procedure will therefore adopt larger stimuli, wider response fields surrounding stimuli, and more response patterns rather than a strict FR 1 contingency to better conform to pigeon peck responses.

Another interesting use of this procedure is to investigate the role of behavioral variability and stereotypy on insight, as some authors have recognized a strong relation between them (Blaisdell et al., 2016; Silva Rodrigues & Garcia‐Mijares, 2025). One way of achieving this is by presenting different groups of pigeons with different functional properties associated with the same stimuli (e.g., a portal that can be used to move the pacman through barriers or thrown to pull the banana toward the pacman). In this case, one group would be presented with only the stimuli having the same functional properties across trials (stereotypy) and another group with the stimuli having different functional properties across trials (variability). Then, the performances of these two groups could be compared within an identical problem, thus investigating the relation between behavioral variability and problem‐solving behavior. Another future direction could be to use this procedure to replicate Luciano (1991) in which pigeons that were trained to push boxes away from a green spot were unable to show the insightful behavior established in Epstein et al. (1984).

Development of a pacman navigation task opens the possibility of studying many other types of psychological and behavioral phenomena. For example, navigation through virtual mazes could be used to study small‐scale navigation, spatial inference, and route planning. Our virtual‐navigation task also lends itself to use with other species that can readily use a touchscreen such as chimpanzees, gorillas, and Japanese macaques (Huskisson et al., 2020); capuchin monkeys (Brando et al., 2021); rats (Cook et al., 2004); crows (Moll & Nieder, 2014); grackles (Seitz et al., 2021); and humans (Gashaj et al., 2021; Huber et al., 2016). Thus, by using a single procedure with a variety of species, we gain a better understanding of the behavioral and possibly cognitive processes that are behind problem‐solving and insight in animals.

## AUTHOR CONTRIBUTIONS

RR contributed to conceptualization, data curation, formal analysis, funding acquisition, investigation, methodology, project administration, validation, visualization, writing original draft, review, and revisions. CK contributed to data curation, formal analysis, methodology, project administration, software, validation, writing original draft, review, and revisions. MM contributed to conceptualization, project administration, resources, supervision, validation, visualization, writing original draft, review, and revisions. AB contributed to conceptualization, funding acquisition, methodology, project administration, resources, software, supervision, validation, visualization, original draft, review, and revisions.

## CONFLICT OF INTEREST STATEMENT

The authors declare no conflicts of interest.

## ETHICS APPROVAL

This research was conducted following the relevant ethics guidelines for research with animals, was approved by UCLA's institutional IACUC, and was in compliance with the APA ethical standards in the treatment of animals.

## Supporting information


**Table S1** Training Phase 1 initial training subphases summary of group results.
**Table S2** Training Phase 1 quiz subphase group results summary.
**Table S3** Training Phase 1 summary of group results (quantitative measures).
**Table S4** Training Phase 2 summary of group results (quantitative measures).
**Table S5** Insight Test performance across Puzzles for each pigeon.

## Data Availability

The data sets generated and analyzed during the current study are available on figshare.

## References

[jeab70083-bib-0001] Birch, H. (1945). The relation of previous experience to insightful problem solving. Journal of Comparative Psychology, 38(6), 367–383. 10.1037/h0056104 21010765

[jeab70083-bib-0002] Blaisdell, A. P. (2009). The role of associative processes in spatial, temporal, and causal cognition. In S. Watanabe , A. P. Blaisdell , L. Huber , & A. Young (Eds.), Rational animals, irrational humans (pp. 153–172). Keio University.

[jeab70083-bib-0003] Blaisdell, A. P. (2019). Mental imagery in animals: Learning, memory, and decision making in the face of missing information. Learning & Behavior, 47(3), 193–216. 10.3758/s13420-019-00386-5. 31228005

[jeab70083-bib-0004] Blaisdell, A. P. , Stolyarova, A. , & Stahlman, W. D. (2016). The law of expect or a modified law of effect? Conductual, 4(2), 61–90. 10.59792/VEEC8896

[jeab70083-bib-0005] Brando, S. , Basom, L. , Bashaw, M. , Druyor, C. , Fonte, E. , & Thompson, R. (2021). Individualized target training facilitated transfer of group housed capuchin monkeys (*Sapajus apella*) to test cubicles and discrimination of targets on computer touch screens. Animals, 11(7), Article 2070. 10.3390/ani11072070. 34359198 PMC8300241

[jeab70083-bib-0006] Call, J. (2013). Three ingredients for becoming a creative tool user. In C. Boesch , C. M. Sanz , & J. Call (Eds.), Tool use in animals: Cognition and ecology (pp. 3–20). Cambridge University Press.

[jeab70083-bib-0007] Chen, M. K. , & Risen, J. L. (2010). How choice affects and reflects preferences: revisiting the free‐choice paradigm. Journal of Personality and Social Psychology, 99(4), 573–594. 10.1037/a0020217 20658837

[jeab70083-bib-0008] Cook, R. G. , & Fowler, C. (2014). “Insight” in pigeons: Absence of means‐end processing in displacement tests. Animal Cognition, 17, 207–220. 10.1007/s10071-013-0653-8 23774955 PMC3894253

[jeab70083-bib-0009] Cook, R. G. , Geller, A. I. , Zhang, G. R. , & Gowda, R. A. M. (2004). Touchscreen‐enhanced visual learning in rats. Behavior Research Methods, Instruments, & Computers, 36(1), 101–106. 10.3758/BF03195555 15190705

[jeab70083-bib-0010] Davis, G. A. (1966). Current status of research and theory in human problem solving. Psychological Bulletin, 66(1), 36–54. 10.1037/h0023460 5329603

[jeab70083-bib-0011] Duncker, K. , & Lees, L. S. (1945). On problem‐solving. Psychological Monographs, 58(5), 1–13. 10.1037/h0093599

[jeab70083-bib-0012] Dwyer, D. M. , & Burgess, K. V. (2011). Rational accounts of animal behaviour? Lessons from C. Lloyd Morgan's Canon. International Journal of Comparative Psychology, 24(4), 349–364. 10.46867/ijcp.2011.24.04.05

[jeab70083-bib-0013] Epstein, R. (1985a). Animal cognition as the praxist views it. Neuroscience & Biobehavioral Reviews, 9(4), 623–630. 10.1016/0149-7634(85)90009-0 3909017

[jeab70083-bib-0014] Epstein, R. (1985b). The spontaneous interconnection of three behaviors. The Psychological Record, 35, 131–141. 10.1007/BF03394917

[jeab70083-bib-0015] Epstein, R. (1987). The spontaneous interconnection of four repertoires of behavior in a pigeon (*Columba livia*). Journal of Comparative Psychology, 101(2), 197–201. 3608425

[jeab70083-bib-0016] Epstein, R. , Kirshnit, C. E. , Lanza, R. P. , & Rubin, L. (1984). ‘Insight’ in the pigeon: Antecedents and determinants of an intelligent performance. Nature, 308(5954), 61–62. 10.1038/308061a0 6700713

[jeab70083-bib-0017] Galinsky, A. D. , & Moskowitz, G. B. (2000). Counterfactuals as behavioral primes: Priming the simulation heuristic and consideration of alternatives. Journal of Experimental Social Psychology, 36(4), 384–409. 10.1006/jesp.1999.1409

[jeab70083-bib-0018] Gashaj, V. , Dapp, L. C. , Trninic, D. , & Roebers, C. M. (2021). The effect of video games, exergames and board games on executive functions in kindergarten and 2nd grade: An explorative longitudinal study. Trends in Neuroscience and Education, 25, Article 100162. 10.1016/j.tine.2021.100162 34844694

[jeab70083-bib-0019] Guttman, N. , & Kalish, H. I. (1956). Discriminability and stimulus generalization. Journal of Experimental Psychology, 51(1), 79–88. 10.1037/h0046219 13286444

[jeab70083-bib-0020] Harris C. R. , Millman K. J. , van der Walt S. J. , Gommers R. , Virtanen P. , Cournapeau D. , Wieser E. , Taylor J. , Berg S. , Smith N. J. , Kern R. , Picus M. , Hoyer S. , van Kerkwijk M. H. , Brett M. , Haldane A. , Del Río J. F. , Wiebe M. , Peterson P. , … Oliphant T.E. (2020). Array programming with NumPy. Nature, 585, 357–362. 10.1038/s41586-020-2649-2 32939066 PMC7759461

[jeab70083-bib-0021] Hayes, S. C. , & Brownstein, A. J. (1986). Mentalism, behavior‐behavior relations, and a behavior‐analytic view of the purposes of science. The Behavior Analyst, 9, 175–190. 10.1007/BF03391944 22478660 PMC2741891

[jeab70083-bib-0022] Holth, P. (2008). What is a problem? Theoretical conceptions and methodological approaches to the study of problem solving. European Journal of Behavior Analysis, 9(2), 157–172. 10.1080/15021149.2008.11434302

[jeab70083-bib-0023] Huber, B. , Tarasuik, J. , Antoniou, M. N. , Garrett, C. , Bowe, S. J. , Kaufman, J. , & Team, S. B. (2016). Young children's transfer of learning from a touchscreen device. Computers in Human Behavior, 56, 56–64. 10.1016/j.chb.2015.11.010

[jeab70083-bib-0024] Hull, C. L. (1939). The problem of stimulus equivalence in behavior theory. Psychological Review, 46(1), 9–30. 10.1037/h0054032

[jeab70083-bib-0025] Huskisson, S. M. , Jacobson, S. L. , Egelkamp, C. L. , Ross, S. R. , & Hopper, L. M. (2020). Using a touchscreen paradigm to evaluate food preferences and response to novel photographic stimuli of food in three primate species (*Gorilla gorilla gorilla*, *Pan troglodytes*, and *Macaca fuscata*). International Journal of Primatology, 41(1), 5–23. 10.1007/s10764-020-00131-0

[jeab70083-bib-0026] Kirkman, C. F. , Rodrigues, R. S. , Garcia‐Mijares, M. , & Blasidell, A. P. (2025). How to teach pigeons to play Pacman: Virtual 2D spatial navigation using a touchscreen video game. PsychArchives. 10.23668/psycharchives.21213

[jeab70083-bib-0027] Köhler, W. (1925/1959). The mentality of apes ( E. Winter , Trans.). Vintage Books.

[jeab70083-bib-0028] Kounios, J. , & Beeman, M. (2014). The cognitive neuroscience of insight. Annual Review of Psychology, 65, 71–93. 10.1146/annurev-psych-010213-115154 24405359

[jeab70083-bib-0029] Luciano, C. (1991). Problem solving behavior: An experimental example. Psicothema, 3 (2), 297–397.

[jeab70083-bib-0030] Lundh, F. (1999). An introduction to Tkinter. PythonWare. https://ftp.math.utah.edu/u/ma/hohn/linux/tcl/an-introduction-to-tkinter.pdf

[jeab70083-bib-0031] Mann, H. B. , & Whitney, D. R. (1947). On a test of whether one of two random variables is stochastically larger than the other. The Annals of Mathematical Statistics, 18(1), 50–60. http://www.jstor.org/stable/2236101

[jeab70083-bib-0032] Milosavljevic, M. , Malmaud, J. , Huth, A. , Koch, C. , & Rangel, A. (2010). The drift diffusion model can account for the accuracy and reaction time of value‐based choices under high and low time pressure. Judgment and Decision Making, 5(6), 437–449. 10.1017/S1930297500001285

[jeab70083-bib-0033] Miyata, H. , & Fujita, K. (2012). Further tests of pigeons' (*Columba livia*) planning behavior using a computerized plus‐shaped maze task. Perceptual and Motor Skills, 115(1), 27–42. 10.2466/23.04.22.PMS.115.4.27-42 23033743

[jeab70083-bib-0034] Miyata, H. , Ushitani, T. , Adachi, I. , & Fujita, K. (2006). Performance of pigeons (*Columba livia*) on maze problems presented on the LCD screen: In search for preplanning ability in an avian species. Journal of Comparative Psychology, 120(4), Article 358. 10.1037/0735-7036.120.4.358 17115856

[jeab70083-bib-0035] Moll, F. W. , & Nieder, A. (2014). The long and the short of it: Rule‐based relative length discrimination in carrion crows. Corvus corone. Behavioral Processes, 107, 142–149. 10.1016/j.beproc.2014.08.009 25151937

[jeab70083-bib-0036] Murray, M. A. , & Byrne, R. M. (2013). Cognitive change in insight problem solving: Initial model errors and counterexamples. Journal of Cognitive Psychology, 25(2), 210–219. 10.1080/20445911.2012.743986

[jeab70083-bib-0037] Neiworth, J. J. , & Rilling, M. E. (1987). A method for studying imagery in animals. Journal of Experimental Psychology: Animal Behavior Processes, 13(3), 203–214. 10.1037/0097-7403.13.3.203

[jeab70083-bib-0038] Neves Filho, H. B. , Assaz, D. A. , Dicezare, R. H. F. , Knaus, Y. C. , & Garcia‐Mijares, M. (2021). Learning behavioral repertoires with different consequences hinders the interconnection of these repertoires in pigeons in the box displacement test. The Psychological Record, 71(4), 567–575. 10.1007/s40732-020-00407-0

[jeab70083-bib-0039] Neves Filho, H. B. , de Carvalho Neto, M. B. , Taytelbaum, G. P. T. , Malheiros, R. D. S. , & Knaus, Y. C. (2016). Effects of different training histories upon manufacturing a tool to solve a problem: Insight in capuchin monkeys (*Sapajus* spp.). Animal Cognition, 19(6), 1151–1164. 10.1007/s10071-016-1022-1 27509890

[jeab70083-bib-0040] Neves Filho, H. B. , Knaus, Y. C. , & Taylor, A. H. (2019). New Caledonian crows can interconnect behaviors learned in different contexts, with different consequences and after exposure to failure. International Journal of Comparative Psychology, 32, 1–19. 10.46867/ijcp.2019.32.00.11

[jeab70083-bib-0041] Neves Filho, H. B. , Stella, L. D. , Dicezare, R. H. , & Garcia‐Mijares, M. (2015). Insight in the white rat: Spontaneous interconnection of two behaviors in *Rattus norvegicus* . European Journal of Behavior Analysis, 16(2), 188–201. 10.1080/15021149.2015.1083283.

[jeab70083-bib-0042] Osuna‐Mascaró, A. J. , & Auersperg, A. M. I. (2021). Current understanding of the “insight” phenomenon across disciplines. Frontiers in Psychology, 12, Article 791398. 10.3389/fpsyg.2021.791398 34975690 PMC8715918

[jeab70083-bib-0043] Pavlov, I. P. (1932). The reply of a physiologist to psychologists. Psychological Review, 39(2), 91–127. 10.1037/h0069929

[jeab70083-bib-0044] Racey, D. , Young, M. E. , Garlick, D. , Pham, J. N‐M. , & Blaisdell, A. P. (2011). Pigeon and human performance in a multi‐armed bandit task in response to changes in variable interval schedules. Learning & Behavior, 39, 245–258. 10.3758/s13420-011-0025-7 21380732

[jeab70083-bib-0045] Ratcliff, R. , & McKoon, G. (2008). The diffusion decision model: Theory and data for two‐choice decision tasks. Neural computation, 20(4), 873–922. 10.1162/neco.2008.12-06-420 18085991 PMC2474742

[jeab70083-bib-0046] Rescorla, R. A. (1986). Extinction of facilitation. Journal of Experimental Psychology: Animal Behavior Processes, 12(1), 16–24. 10.1037/0097-7403.12.1.16

[jeab70083-bib-0047] Rodrigues, R. S. , & Garcia‐Mijares, M. (2021). To fail or not to fail? Implications of extinction on creativity and problem‐solving behavior. The Psychological Record, 71(4), 525–542. 10.1007/s40732-021-00482-x

[jeab70083-bib-0048] Santana, L. H. , & Garcia‐Mijares, M. (2022). Animal creativity as a function of behavioral innovation and behavior flexibility in problem‐solving situations. Integrative Psychological and Behavioral Science, 56(1), 218–233. 10.1007/s12124-020-09586-5 33733318

[jeab70083-bib-0049] Seitz, B. M. , McCune, K. , MacPherson, M. , Bergeron, L. , Blaisdell, A. P. , & Logan, C. J. (2021). Using touchscreen equipped operant chambers to study animal cognition. Benefits, limitations, and advice. PLoS ONE, 16(2), Article e0246446. 10.1371/journal.pone.0246446 33606723 PMC7894864

[jeab70083-bib-0050] Shettleworth, S. J. (2012). Do animals have insight, and what is insight anyway? Canadian Journal of Experimental Psychology/Revue canadienne de psychologie expérimentale, 66(4), 217–226. 10.1037/a0030674 23231629

[jeab70083-bib-0051] Silva Rodrigues, R. , & Garcia‐Mijares, M. (2025). Fostering creativity: The role of operant variability on problem‐solving and insight. The Psychological Record, 75(1), 83–103. 10.1007/s40732-024-00626-9

[jeab70083-bib-0052] Skinner, B. F. (1984). Representations and misrepresentations. Behavioral and Brain Sciences, 7(4), 655–667. 10.1017/S0140525X00027989

[jeab70083-bib-0053] Spence, K. W. (1936). The nature of discrimination learning in animals. Psychological Review, 43(5), 427–449. 10.1037/h0056975 14912194

[jeab70083-bib-0054] Sturz, B. R. , Bodily, K. D. , & Katz, J. S. (2009). Dissociation of past and present experience in problem solving using a virtual environment. CyberPsychology & Behavior, 12(1), 15–19. 10.1089/cpb.2008.0147 19196044

[jeab70083-bib-0055] Thorndike, E. L. (1898). Animal intelligence: An experimental study of the associative processes in animals. The Psychological Review: Monograph Supplements, 2(4). 10.1037/h0092987

[jeab70083-bib-0056] Toegel, F. , Toegel, C. , & Perone, M. (2021). Design and evaluation of a touchscreen apparatus for operant research with pigeons. Journal of The Experimental Analysis of Behavior, 116(2), 249–264. 10.1002/jeab.707 34236081 PMC8882370

[jeab70083-bib-0057] Turner, C. H. (1909). The behavior of a snake. Science, 30(773), 563–564. 10.1126/science.30.773.563 17817501

[jeab70083-bib-0058] Urcuioli, P. J. (2006). Responses and acquired equivalence classes. In E. A. Wasserman (Ed.), Comparative cognition: Experimental explorations of animal intelligence (pp. 405–424). Oxford University Press.

[jeab70083-bib-0059] Urcuioli, P. J. , Zentall, T. R. , Jackson‐Smith, P. , & Steirn, J. N. (1989). Evidence for common coding in many‐to‐one matching: Retention, intertrial interference, and transfer. Journal of Experimental Psychology: Animal Behavior Processes, 15(3), 264–273. 10.1037/0097-7403.15.3.264

[jeab70083-bib-0060] Wasserman, E. A. , DeVolder, C. L. , & Coppage, D. J. (1992). Non‐similarity‐based conceptualization in pigeons via secondary or mediated generalization. Psychological Science, 3(6), 374–379. 10.1111/j.1467-9280.1992.tb00050.x

[jeab70083-bib-0061] Wessells, M. G. (1982). A critique of Skinner's views on the obstructive character of cognitive theories. Behaviorism, 10(1), 65–84. https://www.jstor.org/stable/27758996

