# Supplementary material for: Pigeons, portals, and Pacman: Insightful problem solving and navigation using a touchscreen video game
Source: J Exp Anal Behav. 2026 Feb 1;125(2):e70083. doi: 10.1002/jeab.70083 (PMC12862048; doi:10.1002/jeab.70083)
Supplement: Supplementary file 1 — Table S1 Training Phase 1 initial training subphases summary of group results. Table S2 Training Phase 1 quiz subphase group results summary. Table S3 Training Phase 1 summary of group results (quantitative measures). Table S4 Training Phase 2 summary of group results (quantitative measures). Table S5 Insight Test performance across Puzzles for each pigeon. [file JEAB-125-0-s001.pdf]

Supplementary Material

1. Detailed Individual and Group Results

Table S1

Training Phase 1 initial training subphases summary of group results

Trial Latency Results (*seconds*)					
Subphase	*Mean latency (first, last)*	*SD (first, last)*	*GLM estimate (Latency ~ Session)*	*SE*	*t*
1A	25.78, 2.58	58.07, 1.55	-4.05	2.78	-1.46
1B	38.88, 3.93	52.78, 2.38	-7.75*	3.24	-2.34
1C	27.61, 7.23	20.42, 3.01	-6.69***	1.69	-3.97
1D	8.90, 5.86	9.36, 1.63	0.42	1.42	0.29
1E	10.83, 5.89	7.17, 0.89	-0.82**	.293	-2.80
1F	37.65, 20.72	7.49, 7.53	-15.50	47.63	-3.25
1G	45.00, 27.11	21.43, 11.65	-3.64	2.28	-1.60
1H	67.40, 45.45	54.61, 26.70	9.46	46.19	0.21
1I	18.61, 13.73	10.91, 4.45	-0.24	0.60	-0.49
Movement Accuracy Results (*0 - 1*)					
Subphase	*Mean accuracy (first, last)*	*SD (first, last)*	*Beta regression estimate (Accuracy ~ Session)*	*SE*	*z*
1F	0.40, 0.65	0.07, 0.18	0.28**	0.46	2.80
1H	0.49, 0.59	0.18, 0.17	-0.01	0.01	-0.73
1I	0.66, 0.80	0.12, 0.08	-0.01	0.02	-0.72

Note. Dependent variables were movement accuracy (wherein only trials with correct directional cursor choices were reinforced) and/or trial latency (wherein all trials were reinforced). Regressions were calculated across subject trial mean data utilizing either a generalized linear model for latency (data reported in seconds) or a Beta regression estimate for accuracy (data bound between 0 and 1). P-values are reported as asterisks (* = 0.05, ** = 0.01, & *** = 0.001), where applicable.

Table S2

Training Phase 1 quiz subphase group results summary

Subphase	Combined SAP Results					RWM Comparisons Results	
	Mean SAP (first, last)	*SD (first, last)*	*GLM estimate (SAP ~ Session)*	*SE*	*t*	*Mean RWM SAP*	*Wilcoxon Rank-Sum Test (W)*
1F Quiz	2.83, 1.39	4.08, 0.68	-0.38**	0.12	-3.20	15.15	43451***
1H Quiz	7.51, 3.03	9.39, 5.42	-0.27***	0.06	-4.20	25.32	14460***
1I Quiz	4.28, 1.06	7.30, 0.14	-0.17***	0.28	-6.33	19.11	31126***
1J Quiz	6.01, 1.21	6.08, 0.64	-0.30**	0.01	-2.99	34.83	8030***
1O Quiz	7.65, 3.94	6.86, 3.14	-0.54**	0.20	-2.69	66.29	928***

Note. The primary quantitative measures for the open-arena quiz subphases were residual steps above par (SAP) and random-walk model (RWM) comparisons. P-values are reported as asterisks (* = 0.05, ** = 0.01, & *** = 0.001), where applicable.

Table S3

Training Phase 1 summary of group results (quantitative measures)

Subphase	SAP (Absolute Data) Results		SAP ~ Session Results			RWM Comparisons Results	
Stat.	*Mean SAP (first, last)*	*SD (first, last)*	*GLM estimate (SAP ~ Session)*	*SE*	*t*	*Mean RWM SAP*	*Wilcoxon Rank-Sum Test (W)*
1K	1.82, 1.50	1.78, 1.25	-.108***	0.03	-3.59	39.43	38900***
1L	3.09, 1.20	1.21, 1.00					
1M	10.18, 2.68	7.81, 1.43	-1.50***	0.29	-5.23	70.16	4542***
1N	10.82, 2.69	5.36, 1.52	-0.42	0.30	-1.44		
1O Quiz	7.65, 3.94	6.86, 3.14	-0.54**	0.20	-2.69	66.29	928***

Note. Some analyses are performed across multiple related subphases (e.g., GLM estimates of SAP ~ Session for 1K and 1L). P-values are reported as asterisks (* = 0.05, ** = 0.01, & *** = 0.001), where applicable.

Table S4

Training Phase 2 summary of group results (quantitative measures)

SAP (Absolute Data) Results		SAP ~ Session Results			Perfect Portal Use (Absolute Data) Results		Perfect Portal Use Trials ~ Session Results		
Mean SAP (first, last)	*SD (first, last)*	*GLM estimate (SAP ~ Session)*	*SE*	*t*	*Mean "perfect" portal use (first, last)*	*SD (first, last)*	*Beta regression estimate (Mean Perfect Portal Use Trials ~ Session)*	*SE*	*z*
6.57, 3.95	3.78, 4.70	-0.35***	0.08	-4.30	37.42, 64.85%	48.55, 47.89	0.14**	0.05	2.83

Note. P-values are reported as asterisks (* = 0.05, ** = 0.01, & *** = 0.001), where applicable.

Table S5

Insight Test performance across Puzzles for each pigeon

Puzzle	Subject	Trials Comp.	First SAP	Mean SAP	SAP ~ Trial	First Latency	Mean Latency	Latency ~ Trial	First IRI	Mean IRI	IRI ~ Trial
I	Herriot	13	4	10.5	0.07	35.34	73.99	2.47	0.67	1.08	0.01
	Wario	11	8	2.4	-0.87*	36.70	30.39	-3.32	0.65	0.83	-0.01
	Yoshi	3	2	22.5	NA	31.18	398.54	NA	1.24	2.35	NA
II	Herriot	12	14	4.67	-0.59	75.09	36.56	-2.37	0.85	0.75	-0.01
	Wario	16	0	2.88	-0.01	11.1	20.24	-1.10	0.41	0.64	-0.01
	Yoshi	4	2	4.00	NA	62.49	48.83	NA	1.69	0.94	NA
III	Herriot	12	4	3.17	-0.51*	41.92	39.30	-1.93	0.82	0.83	0.02
	Wario	16	8	4.44	-0.49	66.40	49.14	-3.11	0.87	1.11	0.01
	Yoshi	2	2	2.00	NA	49.46	53.23	NA	0.91	0.83	NA
IV	Herriot	10	8	24.11	6.50	48.09	118.55	32.50	0.48	0.76	-0.03
	Wario	11	6	14.01	-3.04	25.40	123.50	-20.34	0.43	0.91	0.04
	Yoshi	8	34	6.00	-2.48	277.35	81.72	-15.98	1.13	0.93	-0.05
V	Herriot	10	0	2.30	-0.25	27.53	38.46	1.64	1.07	1.22	0.15
	Wario	17	0	1.41	-0.25	15.90	24.39	-0.96	0.52	0.72	-0.02
	Yoshi	2	2	5.00	NA	46.07	69.62	NA	0.88	0.86	NA

Note. Correlations between variables (y ~ x) are calculated via a generalized linear model, and slopes are shown; significance ($p<0.01$) is reported (*) where applicable. Subjects with fewer than 5 completed trials were excluded from correlation analyses. Correlations include the first trial; means do not. Trial latency and IRI are reported in seconds.

2. Pecking Accuracy Information

One of the major concerns of our procedure was pigeon pecking accuracy on infrared touchscreens, as many of the pecks at navigational guides resulted in impotent "background pecks." During the intervals preceding the selection of the correct navigational guide, the mean peck distance from the center of the guide was 46.54 pixels (SD = 51.64, IQR = 48.12). Given that the radius of the navigational guide was only 15 pixels and adjacent guides were approximately 68.7 pixels away, imprecise pecks were not only impotent but may have substantially interfered with navigational performance as well. Using a two-dimensional kernel density estimation of these specific data, we conservatively estimate that only 16.09% of pecks landed on the correct navigational guide and 41.05% landed within a 30-pixel radius from the center of the correct guide, while 3.05% of pecks resulted in the selection of one of the incorrect guides.
